# Profiling Inhibitor Scaffolds for the Cancer Target Jumonji‐C Domain‐Containing Protein 6

**DOI:** 10.1002/cmdc.202500682

**Published:** 2025-10-31

**Authors:** Thomas P. Corner, Eidarus Salah, Anthony Tumber, James P. Holt‐Martyn, Lennart Brewitz, Christopher J. Schofield

**Affiliations:** ^1^ Chemistry Research Laboratory Department of Chemistry and the Ineos Oxford Institute for Antimicrobial Research University of Oxford 12 Mansfield Road Oxford OX1 3TA UK; ^2^ Present address: Department of Molecular, Cellular, and Developmental Biology Yale University New Haven CT 06511 USA

**Keywords:** 2‐oxoglutarate/α‐ketoglutarate‐dependent oxygenase, bromodomain‐containing protein 4, Desidustat, Enarodustat, high‐throughput inhibition assays, inhibitor discovery, JmjC hydroxylase inhibition, Jumonji‐C domain‐containing protein 6, metastatic castration‐resistant prostate cancer

## Abstract

The human 2‐oxoglutarate‐dependent oxygenase Jumonji‐C domain‐containing protein 6 (JMJD6) catalyzes post‐translational C‐5 lysyl residue hydroxylation in multiple proteins. Aberrant JMJD6 catalysis is associated with the upregulation of androgen receptor splice variant 7 (AR‐V7), which confers resistance towards antiandrogens used for prostate cancer treatment; JMJD6 is thus a promising cancer target. To date, few small‐molecule JMJD6 inhibitors are reported, likely in part reflecting a lack of robust assays to monitor effects of small molecules on catalysis by isolated JMJD6. The use of solid‐phase extraction coupled to mass spectrometry assays is described to screen scaffolds for the development of selective JMJD6 inhibitors. The results reveal that the reported JMJD6 inhibitors WL12, SKLB325, and Compound 7p manifest relatively inefficient JMJD6 inhibition in vitro. By contrast, some, but not all, clinically used inhibitors of the human hypoxia‐inducible factor‐α prolyl hydroxylase domain‐containing proteins (PHDs) efficiently inhibit isolated JMJD6, in particular Enarodustat and Desidustat. The results identify attractive scaffolds for the development of selective, cell permeable JMJD6 inhibitors and suggest that JMJD6 inhibition is a potential off‐target effect of PHD inhibitors in clinical use.

## Graphical abstract

High‐throughput mass spectrometry assays identify scaffolds for the development of inhibitors of the anticancer target Jumonji‐C domain‐containing protein 6 (JMJD6). Molecular docking studies suggest strategies for structure‐guided inhibitor optimization.

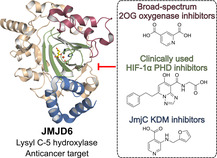

## 1 Introduction

**Figure 1:**
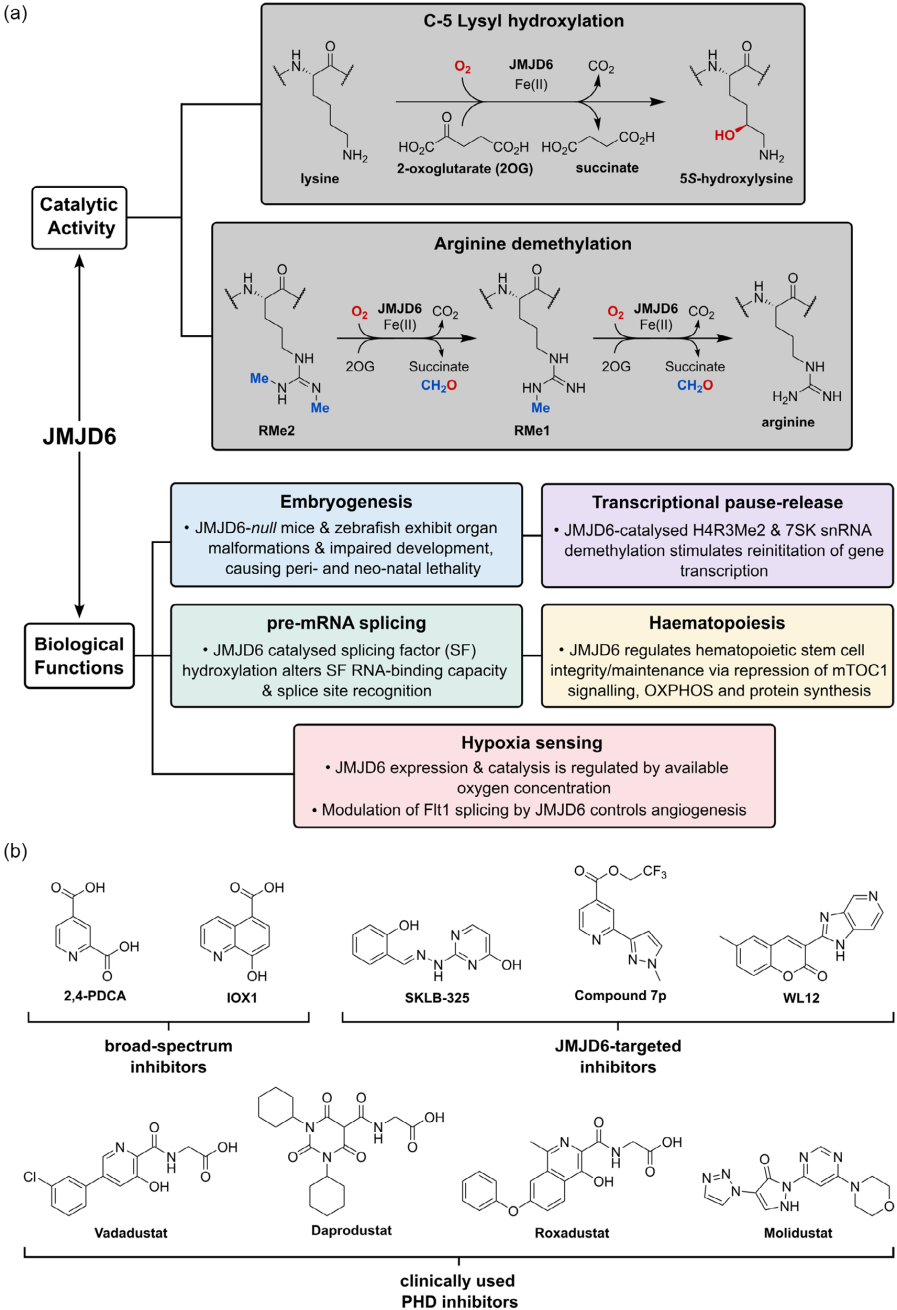
Proposed roles of JMJD6 and selected reported small‐molecule JMJD6 inhibitors. a) JMJD6 catalysis^[^ ^8^ ^]^ and proposed roles in biology.^[^ ^6^ ^,^ ^9^ ^,^ ^12^ ^–^ ^14^ ^]^ b) The reported JMJD6 inhibitors: 2,4‐PDCA,^[^ ^22^ ^]^ IOX1,^[^ ^35^ ^]^ SKLB325,^[^ ^20^ ^]^ Compound 7p,^[^ ^33^ ^]^ WL12,^[^ ^31^ ^,^ ^32^ ^]^ Vadadustat,^[^ ^39^ ^]^ Daprodustat,^[^ ^40^ ^]^ Roxadustat,^[^ ^41^ ^]^ and Molidustat.^[^ ^42^ ^]^

Jumonji‐C domain‐containing protein 6 (JMJD6) is a human 2‐oxoglutarate (2OG)‐ and Fe(II)‐dependent oxygenase that catalyzes the stereospecific post‐translational C‐5 hydroxylation of lysine residues in multiple protein substrates (**Figure** **1**a),^[^ ^1^ ^,^ ^2^ ^]^ including in splicing regulatory (SR) proteins^[^ ^3^ ^,^ ^4^ ^]^ and bromodomain‐containing proteins (BRDs).^[^ ^1^ ^]^ Cellular and biochemical studies have indicated that JMJD6 may also catalyze the *N‐*demethylation of mono‐ and dimethyl arginine residues (Figure 1a);^[^ ^5^ ^,^ ^6^ ^]^ however, not all studies with recombinant JMJD6 have reproduced the assigned demethylation activity.^[^ ^3^ ^,^ ^4^ ^,^ ^7^ ^,^ ^8^ ^]^

JMJD6 is reported to be involved in multiple critical cellular and biological processes,^[^ ^9^ ^]^ including transcription,^[^ ^10^ ^]^ splicing,^[^ ^11^ ^]^ hypoxia sensing,^[^ ^12^ ^]^ hematopoiesis,^[^ ^13^ ^]^ and embryonic development,^[^ ^14^ ^]^ perhaps reflecting its substrate promiscuity.^[^ ^1^ ^,^ ^4^ ^,^ ^9^ ^]^ However, aberrant JMJD6 expression is associated with tumorigeneses and cancer progression.^[^ ^15^, ^16^, ^17^, ^18^, ^19^, ^21^ ^–^ ^22^, ^23^ ^]^ For example, JMJD6 is reported to regulate the generation of the androgen receptor splice variant 7 (AR‐V7), which promotes drug resistance in metastatic castration‐resistant prostate cancer.^[^ ^22^ ^–^ ^24^ ^]^ The combined evidence indicates that inhibition of JMJD6 catalysis may be therapeutically relevant.

Small‐molecule inhibitors of human 2OG oxygenases other than JMJD6 are in clinical use, demonstrating the therapeutic viability of 2OG oxygenase inhibition.^[^ ^25^ ^–^ ^27^ ^]^ For example, inhibitors of the hypoxia‐inducible factor‐α (HIF‐α) prolyl hydroxylase domain‐containing proteins (PHDs) are used for long‐term treatment of chronic kidney disease (CKD)‐associated anemia,^[^ ^27^ ^]^ and Mildronate, an inhibitor of γ‐butyrobetaine hydroxylase (BBOX),^[^ ^28^, ^29^ ^–^ ^30^ ^]^ is used as a cardioprotective agent.^[^ ^26^ ^]^

Despite the apparent therapeutic potential associated with JMJD6 inhibition, relatively few JMJD6‐targeted small‐molecule inhibitors have been reported, including SKLB325,^[^ ^20^ ^]^ WL12,^[^ ^31^ ^,^ ^32^ ^]^ and Compound 7p^[^ ^33^ ^]^ (Figure 1b). SKLB325 is reported to manifest antiproliferative effects in cancer cell lines;^[^ ^20^ ^]^ however, its structural resemblance to the iron chelator pyridoxal isonicotinoyl hydrazone^[^ ^34^ ^]^ raises potential concerns regarding its selectivity and mode of action. The coumarin analogue WL12 is reported to efficiently inhibit JMJD6‐catalyzed histone *N*,*N*‐dimethylarginine demethylation, and cancer cell proliferation.^[^ ^31^ ^,^ ^32^ ^]^ However, mass spectrometry (MS) assays indicated that WL12 does not inhibit JMJD6‐catalyzed lysyl‐residue hydroxylation.^[^ ^31^ ^]^ Although Compound 7p is reported to efficiently and selectively inhibit isolated JMJD6,^[^ ^33^ ^]^ the likely chemical and enzymatic instability of its 2,2,2‐trifluoroethyl ester substituent may limit its cellular and in vivo applications. The broad‐spectrum 2OG oxygenase inhibitor pyridine‐2,4‐dicarboxylic acid (2,4‐PDCA) is also reported to inhibit JMJD6^[^ ^22^ ^,^ ^35^ ^]^ and to induce a dose‐dependent reduction in AR‐V7 protein levels in prostate cancer cells,^[^ ^22^ ^]^ though inhibition of 2OG oxygenases other than JMJD6 may contribute to this effect.^[^ ^22^ ^,^ ^36^, ^37^ ^–^ ^38^ ^]^ JMJD6 is also inhibited by reported PHD inhibitors,^[^ ^35^ ^]^ including clinically used Vadadustat,^[^ ^39^ ^]^ Daprodustat,^[^ ^40^ ^]^ Roxadustat,^[^ ^41^ ^]^ and Molidustat^[^ ^42^ ^]^ (Figure 1b). The combined evidence reveals a need for improved JMJD6‐targeted inhibitors.

The lack of robust high‐throughput inhibition assays of isolated JMJD6 has likely hampered development of JMJD6‐selective inhibitors. Matrix‐assisted laser desorption/ionization time‐of‐flight (MALDI‐TOF) MS‐ and liquid chromatography (LC)MS‐based assays have been used to monitor JMJD6‐catalyzed hydroxylation of a fragment peptide derived from the splicing regulatory protein Luc7‐like 2 (LUC7L2), i.e., LUC7L2_267–278_.^[^ ^22^ ^,^ ^35^ ^]^ However, these assays suffer from relatively low throughput and require relatively high concentrations of JMJD6 (10 μM) and LUC7L2_267–278_ (100 μM). Nuclear magnetic resonance (NMR),^[^ ^35^ ^]^ thermal‐shift,^[^ ^32^ ^,^ ^33^ ^]^ surface plasmon resonance (SPR),^[^ ^20^ ^]^ Succinate‐Glo,^[^ ^31^ ^,^ ^33^ ^]^ and formaldehyde‐coupled detection assays^[^ ^32^ ^]^ have also been employed to identify JMJD6 inhibitors. However, some of these are limited by throughput, do not give a quantitative measure of JMJD6 inhibition, and/or are coupled assays that may be susceptible to compound interference, potentially resulting in identification of false‐positive hits.

Here, we report the use of solid‐phase extraction coupled to mass spectrometry (SPE–MS) assays for inhibition studies with isolated JMJD6. The results identify attractive inhibitor scaffolds for the development of selective, cell permeable small‐molecule JMJD6 inhibitors.

## 2 Results and Discussion

### 2.1 Development of an SPE–MS‐Based JMJD6 Inhibition Assay

SPE–MS assays have been successfully employed to support the development of 2OG oxygenase inhibitors, including for PHD2,^[^ ^43^ ^,^ ^44^ ^]^ factor inhibiting hypoxia inducible factor‐α (FIH),^[^ ^45^ ^,^ ^46^ ^]^ aspartate/asparagine‐β‐hydroxylase (AspH),^[^ ^36^ ^,^ ^47^ ^,^ ^48^ ^]^ BBOX,^[^ ^49^ ^]^ Jumonji‐C domain‐containing protein 5 (JMJD5),^[^ ^50^ ^]^ MYC‐induced nuclear antigen (MINA53),^[^ ^51^ ^]^ fat mass and obesity associated protein (FTO),^[^ ^52^ ^]^ ten‐eleven translation (TET) enzymes,^[^ ^53^ ^,^ ^54^ ^]^ and JmjC histone *N* ^ϵ^‐methyl lysine demethylases 4–6 (KDM4−6).^[^ ^55^ ^]^ Recently, we reported an SPE–MS‐based assay which monitors JMJD6‐catalyzed hydroxylation of a 40mer oligopeptide derived from the bromodomain‐containing protein 4 (BRD4), i.e., BRD4_511–550_,^[^ ^1^ ^,^ ^56^ ^]^ for kinetic studies.^[^ ^56^ ^]^ It was attractive to optimize the SPE–MS assays to investigate effects of small‐molecules on JMJD6 activity, including because: 1) they can be semiautomated using the RapidFire‐associated robotics;^[^ ^36^ ^,^ ^54^ ^,^ ^57^ ^]^ 2) their high sensitivity minimizes enzyme and substrate consumption; and 3) they directly monitor JMJD6 catalyzed BRD4 hydroxylation.

The JMJD6 (0.05 µM) and BRD4_511–550_ (1 µM) SPE–MS assay concentrations were optimized to enable efficient BRD4_511–550_ hydroxylation (enzyme/substrate ratio: 1/20), while avoiding BRD4_511–550_‐substrate induced JMJD6 inhibition, which occurred at concentrations >2 µM.^[^ ^56^ ^]^ The BRD4_511–550_ concentration is close to the JMJD6 $K_{m}^{\mathrm{app}}$ for BRD4_511–550_ (i.e., ≈0.6 µM), and so is consistent with conditions of reported SPE–MS 2OG oxygenase inhibition assays (**Table** **1**).^[^ ^36^ ^,^ ^43^ ^,^ ^50^ ^,^ ^51^ ^,^ ^55^ ^]^

**Table 1:** 2OG oxygenase (co‐)substrate $K_{m}^{\mathrm{app}}$ values and concentrations used in 2OG oxygenase assays. (n.r.: not reported. Note: the oligomeric nature of some of the 2OG oxygenases, including JMJD6 and BBOX, may complicate interpretation of kinetic parameters.).

	Enzymea)	$K_{m}^{\mathrm{app}}$ [µM]			SPE–MS assay concentration [µM]				
		2OG	Fe(II)	Substrateb)	Enzyme	2OG	Fe(II)	Substrate	LAA
i	**JMJD6** c) (ref. [56])	23.3 ± 2.5	0.19 ± 0.02	0.62 ± 0.26	0.05	100	2	1	100
ii	**JMJD5** c) (ref. [50])	0.29 ± 0.04	0.13 ± 0.02	0.87 ± 0.46	0.15	2	10	2	100
iii	**AspH** c) (refs. [36,122])	0.60 ± 0.09	1.42 ± 0.16	1.19 ± 0.26	0.05	3	2	1	100
iv	**FIH** c) (refs. [43,58])	0.8 ± 0.1	n.r.	n.r.	0.15	10	10	5	100
v	**PHD2** d) (refs. [43,123])	0.35 ± 0.03	0.89 ± 0.07	7.3 ± 1.3	0.15	10	10	5	100
vi	**KDM4C** c) (ref. [55])	2.6 ± 0.1	n.r.	5.8 ± 0.7	0.15	10	10	10	100
vii	**BBOX** e) (refs. [49,124,125])	153 ± 44	n.r.	4.2	0.05	400	50	25	500
viii	**MINA53** c) (ref. [51])	3.2 ± 0.6	0.5 ± 0.2	10.5 ± 5.5	0.15	2	50	5	100
ix	**NO66** c) (ref. [51])	0.83 ± 0.09	0.014 ± 0.001	19.1 ± 6.3	0.30	2	50	5	100
x	**FTO** f) (refs. [52,126])	2.9	n.r.	n.r.	0.10	1	0.5	0.6	200
xi	**TET2** g) (ref. [54])	4.9 ± 0.3	n.r.	n.r.	0.30	25	25	1	200

a) Kinetic parameters were determined using.

b) Substrates: BRD4_511–550_ (for JMJD6), RPS6_128–148_ (for JMJD5), hFX‐CP_101–119_ (for AspH), HIF‐1α_789–822_ (for FIH), HIF‐1α‐derived biotin‐DLEMLAPYIPMDDDFQL (for PHD2 kinetics), HIF‐1α_556–574_ (for PHD2 SPE–MS assays), histone 3 K9(me3) derivative ARTAQTARK(me3)STGGIA (for KDM4C), γ‐butyrobetaine (for BBOX), RPL27A_31–49_ (for MINA53), RPL8_205–224_ (for NO66), poly(^14^C‐3‐methylthymine) (for FTO kinetics), AUUGUGG(m^6^A)CUGCAGC (for FTO SPE–MS assays), ACCAC(m^**5**^C)GGTGGT (for TET2).^[^ ^7^ ^,^ ^108^, ^109^, ^110^, ^111^ ^,^ ^113^ ^,^ ^127^ ^]^

c) SPE–MS.

d) Fluorescence resonance energy transfer.

e) ^1^H NMR.

f) Fluorescence‐based assays.

g) AlphaScreen assays.

Co‐substrate concentrations were chosen based on reported SPE–MS kinetic parameters for the JMJD6‐catalyzed BRD4_511–550_ monohydroxylation reaction.^[^ ^56^ ^]^ A 2OG concentration of 100 µM was employed, which is approximately fourfold greater than the $K_{m}^{\mathrm{app}}$(2OG) value of JMJD6 (i.e., ≈25 µM), consistent with reported 2OG oxygenase SPE–MS assays for which the 2OG concentration is typically approximately one‐ to tenfold the 2OG $K_{m}^{\mathrm{app}}$ concentration (Table 1).^[^ ^36^ ^,^ ^43^ ^,^ ^50^ ^–^ ^52^ ^,^ ^54^ ^,^ ^55^ ^,^ ^58^ ^]^ Since JMJD6 has a relatively high 2OG $K_{m}^{\mathrm{app}}$, the JMJD6 SPE–MS inhibition assay employs a 2OG concentration which is relatively high compared to most other 2OG oxygenase SPE–MS assays, possibly more accurately reflecting cellular 2OG concentrations.^[^ ^59^ ^,^ ^60^ ^]^ Likely saturating concentrations of LAA (100 µM) and FAS (2 µM) were used.

JMJD6 catalyzes oxidation of BRD4_511–550_ to give multiple hydroxylation products,^[^ ^56^ ^]^ an observation potentially complicating data analysis. Thus, SPE–MS time‐course studies were performed in 384‐well plates under the optimized conditions (i.e., 0.05 µM JMJD6, 100 µM 2OG, 100 µM LAA, 2 µM Fe(II), 1 µM BRD4_511–550_ in buffer (50 mM Tris, pH 7.5) at ambient temperature) to determine an appropriate assay time such that monohydroxylated BRD4_511–550_ is the dominant product. The results reveal that JMJD6 catalyzed ≈30% monohydroxylation and <5% dihydroxylation of BRD4_511–550_ after 20 min (Figure S1, Supporting Information). The ratio of di‐ to monohydroxylated BRD4_511–550_ increased with reaction times; thus, stopping the JMJD6 reaction at ≈30% BRD4_511–550_ monohydroxylation is preferred in order to ensure that the JMJD6 reaction operates under steady‐state conditions and to simplify data analysis.

### 2.2 Effects of Broad‐Spectrum 2OG Oxygenase Inhibitors on JMJD6 Catalysis

The effects of the reported broad‐spectrum 2OG oxygenase inhibitors 2,4‐PDCA,^[^ ^61^ ^]^ IOX1,^[^ ^62^ ^]^ and *N*‐oxalylglycine (NOG)^[^ ^63^ ^]^ on catalysis of isolated recombinant JMJD6 were investigated (Figure S2, Supporting Information), because these molecules are reported to inhibit JMJD6.^[^ ^22^ ^,^ ^35^ ^]^ Half‐maximal inhibitory concentrations (IC_50_ values) were determined in the presence of DMSO (0.5%_v/v_), which did not substantially affect JMJD6 activity and product ratios (Figure S3, Supporting Information).

The dose response studies reveal 2,4‐PDCA was approximately fourfold more efficient in inhibiting JMJD6 (IC_50_ ≈ 1.6 µM; **Table** **2**, entry i) than the structurally related IOX1 (IC_50_ ≈ 6.4 µM; Table 2, entry ii), while the apparently close 2OG analogue NOG did not affect JMJD6 catalysis in the tested concentration range (IC_50_ > 50 µM; Table 2, entry iii). The SPE–MS IC_50_ value for 2,4‐PDCA was approximately ten‐ and threefold lower than that reported using the MALDI‐TOF MS (IC_50_ ≈ 13 µM)^[^ ^35^ ^]^ and LC–MS (IC_50_ ≈ 5.4 µM)^[^ ^22^ ^]^ assays, respectively. This observation may reflect the different assay conditions and substrate used in the reported MALDI‐TOF MS and LC–MS JMJD6 assays (10 µM JMJD6, 100 µM LUC7L2_267–278_)^[^ ^22^ ^,^ ^35^ ^]^ compared with the SPE–MS assay (0.05 µM JMJD6, 1 µM BRD4_511,550_). By contrast to 2,4‐PDCA, IOX1 inhibited JMJD6 with similar potency in both the SPE–MS and the reported MALDI‐TOF MS assays (IC_50_ ≈ 10 µM).^[^ ^35^ ^]^

**Table 2:** Effects of broad‐spectrum 2OG oxygenase inhibitors, TCA cycle intermediates and structurally related metabolites on JMJD6 catalysis.

	Compound	Structure	IC_50_ [µM]a,, b,, c)		Compound	Structure	IC_50_ [µM]a,, b,, c)
i	2,4‐PDCA^[^ ^61^ ^]^	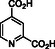	1.6 ± 0.1 (reported: 13^[^ ^35^ ^]^/5.4^[^ ^22^ ^]^)	ix	Isocitrate	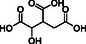	>50 (reported: 1055^[^ ^35^ ^]^)
ii	IOX1^[^ ^62^ ^]^	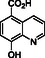	6.4 ± 0.2 (reported: 10^[^ ^35^ ^]^)	x	Succinate	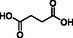	>50 (reported: 261^[^ ^35^ ^]^)
iii	NOG^[^ ^63^ ^]^	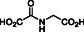	>50 (reported: 296^[^ ^35^ ^]^)	xi	Fumarate	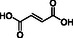	>50 (reported: 165^[^ ^35^ ^]^)
iv	Daminozide^[^ ^64^ ^]^	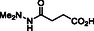	>50 (reported: 94^[^ ^35^ ^]^)	xii	Malate	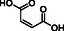	>50 (reported: 5103^[^ ^35^ ^]^)
v	**1** ^[^ ^65^ ^]^	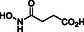	3.6 ± 0.7 (reported: 25^[^ ^35^ ^]^)	xiii	Oxaloacetate	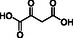	>50 (reported: 204^[^ ^35^ ^]^)
vi	Ebselen^[^ ^66^ ^]^	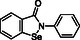	>50	xiv	Mesaconate	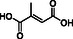	>50
vii	Pyruvate	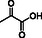	>50 (reported: 2377^[^ ^35^ ^]^)	xv	*D*‐2‐Hydroxyglutarate (*D*‐2HG)	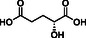	>50 (reported: 622^[^ ^35^ ^]^)
viii	Citrate	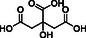	>50 (reported: 987^[^ ^35^ ^]^)	xvi	*L*‐2‐Hydroxyglutarate (*L*‐2HG)	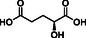	>50 (reported: 871^[^ ^35^ ^]^)

a) SPE–MS inhibition assays were performed as described in the Experimental Section, using full‐length His_6_‐JMJD6 (0.05 µM), 2OG (100 µM), (NH_4_)_2_Fe(SO_4_)_2_·6H_2_O (FAS; 2 µM), BRD4_511–550_ (1 µM), and *L*‐ascorbic acid (100 µM).

b) Means of two independent runs, each composed of technical duplicates (*n* = 2; mean ± SD).

c) Reported IC_50_ values are in parentheses.

The effects of the plant‐growth regulator Daminozide,^[^ ^64^ ^]^ hydroxamic acid **1**,^[^ ^65^ ^]^ and Ebselen^[^ ^66^ ^]^ on JMJD6 activity were investigated because these compounds are reported to inhibit a subset of 2OG oxygenases (Table 2, entries iv–vi).^[^ ^36^ ^,^ ^50^ ^,^ ^64^ ^,^ ^65^ ^,^ ^67^ ^]^ The results reveal that **1** inhibited JMJD6 with moderate efficiency (IC_50_ ≈ 3.6 µM), whereas Daminozide did not inhibit JMJD6 (IC_50_ > 50 μM), the latter observation being consistent with reported MALDI‐TOF MS results.^[^ ^35^ ^]^ Although Ebselen is an efficient inhibitor of both AspH and JMJD5,^[^ ^36^ ^,^ ^50^ ^]^ it did not inhibit JMJD6 within the tested concentration range (IC_50_ > 50 μM), possibly reflecting the absence of solvent‐exposed cysteines in the catalytic JmjC domain of JMJD6 (amino acids 141—305).^[^ ^4^ ^]^

Tricarboxylic acid (TCA) cycle intermediates, the oncometabolite *D*‐2‐hydroxyglutarate (*D*‐2HG), and its enantiomer *L*‐2HG were tested for JMJD6 inhibition because some of them are reported to inhibit some, but not all, human 2OG oxygenases.^[^ ^68^, ^69^, ^70^, ^71^, ^72^, ^73^, ^74^, ^75^, ^76^, ^77^, ^78^, ^79^, ^80^ ^–^ ^81^ ^]^ The results reveal that none of these metabolites affected JMJD6 catalysis in the tested concentration range (IC_50_ > 50 µM; Table 2, entries vii‐xvi), consistent with reported MALDI‐TOF MS inhibition studies.^[^ ^35^ ^]^

### 2.3 Reported JMJD6‐Selective Inhibitors Lack Efficiency in SPE–MS Assays

The SPE–MS assay was then employed to investigate the effects of the reported JMJD6‐selective small‐molecule inhibitors WL12,^[^ ^31^ ^,^ ^32^ ^]^ SKLB325,^[^ ^20^ ^]^ and Compound 7p^[^ ^33^ ^]^ on catalysis of isolated recombinant JMJD6 (**Table** **3**). Notably, the results reveal that WL12 did not affect JMJD6‐catalyzed BRD4_511–550_ hydroxylation (IC_50_ > 50 µM; Table 3, entry i). Although reported fluorescence‐based assays indicated WL12 is a potent inhibitor of JMJD6‐catalyzed histone dimethylarginine *N‐*demethylation (reported IC_50_ ≈ 0.22 µM),^[^ ^32^ ^]^ our observation that WL12 does not inhibit JMJD6‐catalyzed lysine hydroxylation is in accord with reported results obtained with Succinate‐Glo and MALDI‐TOF assays.^[^ ^31^ ^]^ Given that the dimethylarginine *N*‐demethylation activity of JMJD6 has not been consistently observed^[^ ^3^ ^,^ ^4^ ^,^ ^7^ ^,^ ^8^ ^]^ and that coumarin derivatives may interfere with fluorescence‐based enzyme assays, the combined evidence suggests that WL12 is not an efficient inhibitor of purified JMJD6, at least with our hydroxylation assay conditions. Cellular studies with WL12, however, evidence effects on histone H4 Arg‐3 methylation;^[^ ^32^ ^]^ it is possible that WL12 is metabolized to a JMJD6 inhibitor or that its effects on JMJD6 or H4 Arg‐3 methylation status are not mediated (directly) via JMJD6 inhibition.

**Table 3:** Effects of JMJD6‐selective inhibitors on JMJD6 catalysis.

	Compound	Structure	IC_50_ [µM]^a^^,^ b ^,^ c)		Compound	Structure	IC_50_ [µM]^a^^,^ b ^,^ c)
i	WL12^[^ ^32^ ^]^	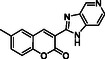	>50 (reported: 0.22^[^ ^32^ ^]^/>100^[^ ^31^ ^]^)	iii	Compound 7*p* ^[^ ^33^ ^]^	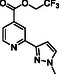	4.2 ± 0.2 (reported: 0.68^[^ ^33^ ^]^)
ii	SKLB325^[^ ^20^ ^]^	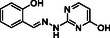	6.7 ± 0.6 (reported: 0.78^[^ ^20^ ^]^)	iv	Compound 10a^[^ ^33^ ^]^	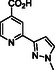	0.07 ± 0.01 (reported: 1.7^[^ ^33^ ^]^)

a) SPE–MS inhibition assays were performed as described in the Experimental Section, using full‐length His_6_‐JMJD6 (0.05 µM), 2OG (100 µM), (NH_4_)_2_Fe(SO_4_)_2_·6H_2_O (FAS; 2 µM), BRD4_511–550_ (1 µM), and *L*‐ascorbic acid (100 µM).

b) Means of two independent runs, each composed of technical duplicates (*n* = 2; mean ± SD).

c) Reported IC_50_ values are in parentheses.

Both SKLB325^[^ ^20^ ^]^ and Compound 7p^[^ ^33^ ^]^ inhibited JMJD6 under the SPE‐MS assay conditions (IC_50_ ≈ 6.7 and 4.2 µM, respectively; Table 3, entries ii–iii), albeit with IC_50_ values approximately nine‐ and sixfold less potent than those reported. The conditions employed to determine the reported JMJD6 IC_50_ value for SKLB325 were, to our knowledge, not described.^[^ ^20^ ^]^ Succinate‐Glo assays were used previously to measure the JMJD6 inhibitory activity of Compound 7p;^[^ ^33^ ^]^ these assays employed a substrate other than BRD4_511–550_ and lower 2OG (10 μM), but higher JMJD6 (10 µM) concentrations than our SPE–MS assay, which likely affects inhibition potency.

The 4‐carboxylic acid analogue of Compound 7p (i.e., Compound 10a^[^ ^33^ ^]^) was tested for JMJD6 inhibition because of the structural similarities between Compound 7p and 2,4‐PDCA. Compound 10a was ≈60‐fold more potent than Compound 7p in inhibiting JMJD6, with an IC_50_ value near the lower intrinsic limit of the SPE–MS assay (IC_50_ ≈ 0.07 µM; Table 3, entry iv). This observation suggests that hydrolysis of Compound 7p to give Compound 10a in cells/under assay conditions may, at least in part, account for the discrepancy between its reported inhibitory activity and our SPE–MS results. The JMJD6 IC_50_ value of Compound 10a was ≈1.7 μM using the Succinate‐Glo™ assay,^[^ ^33^ ^]^ which is ≈25‐fold less potent than that measured using the SPE–MS assay, highlighting the utility of SPE‐MS for inhibitor identification and development. The excellent potency of Compound 10a for JMJD6 inhibition indicates its potential to serve as a lead for the development of improved JMJD6 inhibitors. Note, however, that its mode of inhibition is unknown, though it likely involves active site Fe(II) chelation.

### 2.4 Effects of Selected Reported JmjC KDM Inhibitors on JMJD6 Catalysis

The effects of selected reported JmjC KDM inhibitors on catalysis by isolated JMJD6 were investigated using SPE–MS because of their structural similarity with reported JMJD6 inhibitors (**Table** **4**; entries i–viii). The results reveal that JMJD histone demethylase inhibitor III^[^ ^82^ ^]^ inhibited JMJD6 approximately tenfold more efficiently than 2,4‐PDCA (IC_50_ ≈ 0.10 µM; Table 3, entry i). The methyl ester prodrug of JMJD histone demethylase inhibitor III (i.e., Methylstat^[^ ^82^ ^]^) is commercially available and may thus be useful to investigate the phenotypes of JMJD6 inhibition in cells; however, it should be noted that JMJD histone demethylase inhibitor III also efficiently inhibits 2OG oxygenases other than JMJD6,^[^ ^50^ ^,^ ^82^ ^]^ which may complicate the interpretation of cellular results. Thus, JMJD histone demethylase inhibitor III should be regarded as a broad‐spectrum 2OG oxygenase inhibitor.

**Table 4:** Effects of (partly) selective 2OG oxygenase inhibitors on JMJD6 catalysis.

	Compound	Structure	IC_50_ [µM]^a^^,^ b ^,^ c)		Compound	Structure	IC_50_ [µM]^a^^,^ b ^,^ c)
i	JMJD histone demethylase inhibitor III^[^ ^82^ ^]^	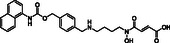	0.10 ± 0.01	xiii	Enarodustat^[^ ^96^ ^]^	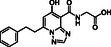	1.2 ± 0.1
ii	ML324^[^ ^83^ ^]^	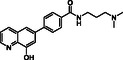	1.4 ± 0.1	xiv	Desidustat^[^ ^97^ ^]^	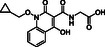	2.7 ± 0.4
iii	Compound 34^[^ ^84^ ^]^	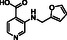	2.6 ± 0.1	xv	Compound 48^[^ ^49^ ^]^	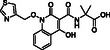	19 ± 4
iv	QC6352^[^ ^86^ ^]^	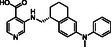	2.8 ± 0.2	xvi	IOX2^[^ ^101^ ^]^	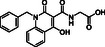	7.1 ± 1.3
v	AS‐8351^[^ ^87^ ^]^	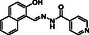	6.9 ± 1.0	xvii	Daprodustat^[^ ^40^ ^]^	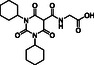	28 ± 2 (reported: 10^[^ ^35^ ^]^)
vi	CPI‐455^[^ ^88^ ^]^	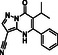	>50	xviii	Vadadustat^[^ ^39^ ^]^	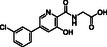	17 ± 1 (reported: 7^[^ ^35^ ^]^)
vii	KDM5A‐IN‐1^[^ ^89^ ^]^	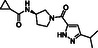	>50	xix	Molidustat^[^ ^42^ ^]^	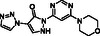	20 ± 1 (reported: 74^[^ ^35^ ^]^)
viii	GSK‐J1^[^ ^90^ ^]^	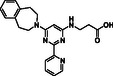	>50	xx	Roxadustat^[^ ^41^ ^]^	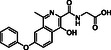	>50 (reported: 23^[^ ^35^ ^]^)
ix	Compound 42^[^ ^45^ ^]^	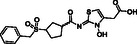	0.37 ± 0.01	xxi	JNJ‐42041935^[^ ^102^ ^]^	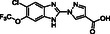	>50
x	BNS^[^ ^92^ ^,^ ^93^ ^]^	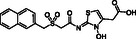	0.16 ± 0.02	xxii	MK‐8617^[^ ^103^ ^]^	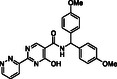	>50
xi	NOFD^[^ ^94^ ^]^	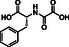	>50	xxiii	IOX5^[^ ^44^ ^]^	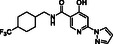	>50
xii	TP0463518^[^ ^95^ ^]^	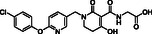	1.1 ± 0.1				

a) SPE–MS inhibition assays were performed as described in the Experimental Section, using full‐length His_6_‐JMJD6 (0.05 µM), 2OG (100 µM), (NH_4_)_2_Fe(SO_4_)_2_·6H_2_O (FAS; 2 µM), BRD4_511–550_ (1 µM), and *L*‐ascorbic acid (100 µM).

b) Means of two independent runs, each composed of technical duplicates (*n* = 2; mean ± SD).

c) Reported IC_50_ values are in parentheses.

The KDM4 inhibitor ML324^[^ ^83^ ^]^ inhibited JMJD6 with similar potency as 2,4‐PDCA (IC_50_ ≈ 1.4 µM; Table 3, entry ii), while the KDM4/5‐selective inhibitor Compound 34^[^ ^84^ ^]^ was approximately twofold less potent (IC_50_ ≈ 2.6 µM; Table 3, entry iii). The observation that Compound 34 inhibits JMJD6 is consistent with reported chemoproteomic pull‐down studies that observed its binding to JMJD6 in cell lysates.^[^ ^85^ ^]^ The KDM4 inhibitor QC6352,^[^ ^86^ ^]^ which is structurally related to Compound 34, manifested similar levels of JMJD6 inhibition (IC_50_ ≈ 2.8 µM; Table 3, entry iv).

The reported KDM5 inhibitor AS‐8351^[^ ^87^ ^]^ manifested moderate levels of JMJD6 inhibition (IC_50_ ≈ 6.9 µM; Table 3, entry v), whereas the KDM5 subfamily‐selective inhibitors CPI‐455^[^ ^88^ ^]^ and KDM5A‐IN‐1,^[^ ^89^ ^]^ and the KDM6 inhibitor GSK‐J1^[^ ^90^ ^]^ did not affect JMJD6 catalysis (IC_50_ > 50 µM; Table 3, entry vi–viii).

### 2.5 Clinically used PHD Inhibitors Inhibit JMJD6

Crystallographic studies have revealed structural similarities between the 2OG binding pockets of JMJD6, FIH, and the PHDs,^[^ ^91^ ^]^ and MALDI‐TOF MS assays have shown that the clinically used PHD inhibitors Roxadustat,^[^ ^41^ ^]^ Daprodustat,^[^ ^40^ ^]^ Molidustat,^[^ ^42^ ^]^ and Vadadustat^[^ ^39^ ^]^ inhibit JMJD6.^[^ ^35^ ^]^ Thus, we investigated the effects of FIH and PHD inhibitors on JMJD6 catalysis using SPE–MS (Table 4, entries ix–xxiii), including a wider panel of clinically used PHD inhibitors and structurally related compounds than previously tested.^[^ ^35^ ^]^

The results reveal that the FIH inhibitor Compound 42^[^ ^45^ ^]^ inhibited JMJD6 with similar potency as it did FIH (IC_50_ ≈ 0.37 µM; Table 4, entry ix). Note that Compound 42 is reported to inhibit PHD2, AspH, and JMJD5 at least an order of magnitude less efficiently than FIH (and JMJD6, as observed here) and to not inhibit KDM4A.^[^ ^45^ ^]^ BNS,^[^ ^92^ ^,^ ^93^ ^]^ which is an *N*‐hydroxythiazole like Compound 42 and which is reported to efficiently inhibit FIH, PHDs, and other human 2OG oxygenases, inhibited JMJD6 approximately tenfold more efficiently than 2,4‐PDCA (IC_50_ ≈ 0.16 µM; Table 4, entry x) and approximately twofold more efficiently than Compound 42, an observation that supports the assignment of BNS as a broad‐spectrum 2OG oxygenase inhibitor. By contrast, the FIH‐selective inhibitor *N*‐oxalyl‐*D*‐phenylalanine (NOFD)^[^ ^94^ ^]^ did not inhibit JMJD6 (IC_50_ > 50 µM; Table 4, entry xi). The combined results thus indicate that the *N*‐hydroxythiazole scaffold is suitable for the development of PHD‐, FIH‐ and/or JMJD6‐targeting inhibitors.

The results also reveal that several of the tested PHD inhibitors were efficient JMJD6 inhibitors, in particular TP0463518^[^ ^95^ ^]^ (IC_50_ ≈ 1.1 µM), Enarodustat^[^ ^96^ ^]^ (IC_50_ ≈ 1.2 µM), and Desidustat^[^ ^97^ ^]^ (IC_50_ ≈ 2.7 µM), which, to our knowledge, were not previously tested for JMJD6 inhibition. This observation is of interest because both Enarodustat^[^ ^98^ ^]^ and Desidustat^[^ ^97^ ^]^ are clinically used to treat CKD‐associated anemia and because TP0463518 is under clinical evaluation.^[^ ^99^ ^]^ Both Enarodustat^[^ ^96^ ^]^ and Desidustat^[^ ^97^ ^]^ are reported to manifest selectivity for inhibition of isolated PHD2 over other isolated 2OG oxygenases (e.g., AspH, FIH, KDM4A, JMJD5, and MINA53),^[^ ^100^ ^]^ with the exception of BBOX.^[^ ^49^ ^]^

Although the BBOX inhibitor Compound 48^[^ ^49^ ^]^ and the PHD inhibitor IOX2^[^ ^101^ ^]^ are structurally related to Desidustat, they inhibited JMJD6 substantially less efficiently than Desidustat (IC_50_ ≈ 7.1 and ≈19 µM, respectively; Table 4, entries xv–xvi). Compound 48 thus exhibits approximately 1000‐fold selectivity for BBOX over JMJD6 inhibition, supporting its suitability as a chemical probe to investigate the cellular phenotypes of BBOX inhibition^[^ ^49^ ^]^ and the suitability of the Desidustat scaffold for the design of (partly) selective 2OG oxygenase inhibitors.

In contrast with the results for TP0463518, Enarodustat and Desidustat, all the other tested clinically used PHD inhibitors Daprodustat^[^ ^40^ ^]^ (IC_50_ ≈ 28 µM), Vadadustat^[^ ^39^ ^]^ (IC_50_ ≈ 17 µM), Molidustat^[^ ^42^ ^]^ (IC_50_ ≈ 20 µM), and, in particular, Roxadustat^[^ ^41^ ^]^ (IC_50_ > 50 µM) manifested relatively inefficient JMJD6 inhibition: they inhibited JMJD6 at least an order of magnitude less efficiently than Enarodustat (Table 4, entries xvii–xx). The JMJD6 IC_50_ values for Daprodustat and Vadadustat were approximately threefold greater than those reported using MALDI‐TOF MS assays, whilst the IC_50_ value for Molidustat was approximately fourfold lower than that reported using MALDI‐TOF MS, potentially reflecting the different assay conditions and substrates used. The observation that Daprodustat, Roxadustat, Vadadustat, and Molidustat manifest comparatively inefficient JMJD6 inhibition is consistent with reported studies, showing that they have no substantial effects on catalysis of 2OG oxygenases other than the PHDs,^[^ ^25^ ^,^ ^36^ ^,^ ^45^ ^,^ ^50^ ^]^ with the exception of Vadadustat which typically manifested lower selectivity levels.^[^ ^36^ ^,^ ^49^ ^]^

The PHD inhibitors JNJ‐42041935,^[^ ^102^ ^]^ MK‐8617,^[^ ^103^ ^]^ and IOX5^[^ ^44^ ^]^ did not inhibit JMJD6 in the tested concentration range (IC_50_s > 50 µM; Table 4, entries xxi–xxiii). Both MK‐8617^[^ ^103^ ^]^ and IOX5^[^ ^44^ ^]^ are proposed to primarily inhibit the PHDs by binding to their HIF‐α substrate pocket, suggesting that substrate‐competitive inhibition may be a promising strategy to achieve selectivity for PHD over JMJD6 inhibition.

### 2.6 Computationally Predicted Binding Modes of Selected JMJD6 Inhibitors

Protein‐ligand docking studies based on crystal structures were performed to investigate the potential JMJD6 binding modes of a structurally relatively diverse subset of the most efficient JMJD6 inhibitors identified in this work (i.e., Compound 10a,^[^ ^33^ ^]^ BNS,^[^ ^92^ ^,^ ^93^ ^]^ Enarodustat,^[^ ^96^ ^]^ JMJD histone demethylase inhibitor III,^[^ ^82^ ^]^ Compound 34^[^ ^84^ ^]^) to guide future structure‐based optimization. Docking studies were performed using GOLD 5.1,^[^ ^104^ ^]^ employing a reported JMJD6:Fe:2OG complex cocrystal structure (PDB:6GDY^[^ ^4^ ^]^) as the receptor model. The side‐chain conformations of Y131 and F133 were allowed to be flexible for the docking simulations because they are observed to adopt different conformations in JMJD6 crystal structures.^[^ ^4^ ^,^ ^105^ ^,^ ^106^ ^]^

**Figure 2:**
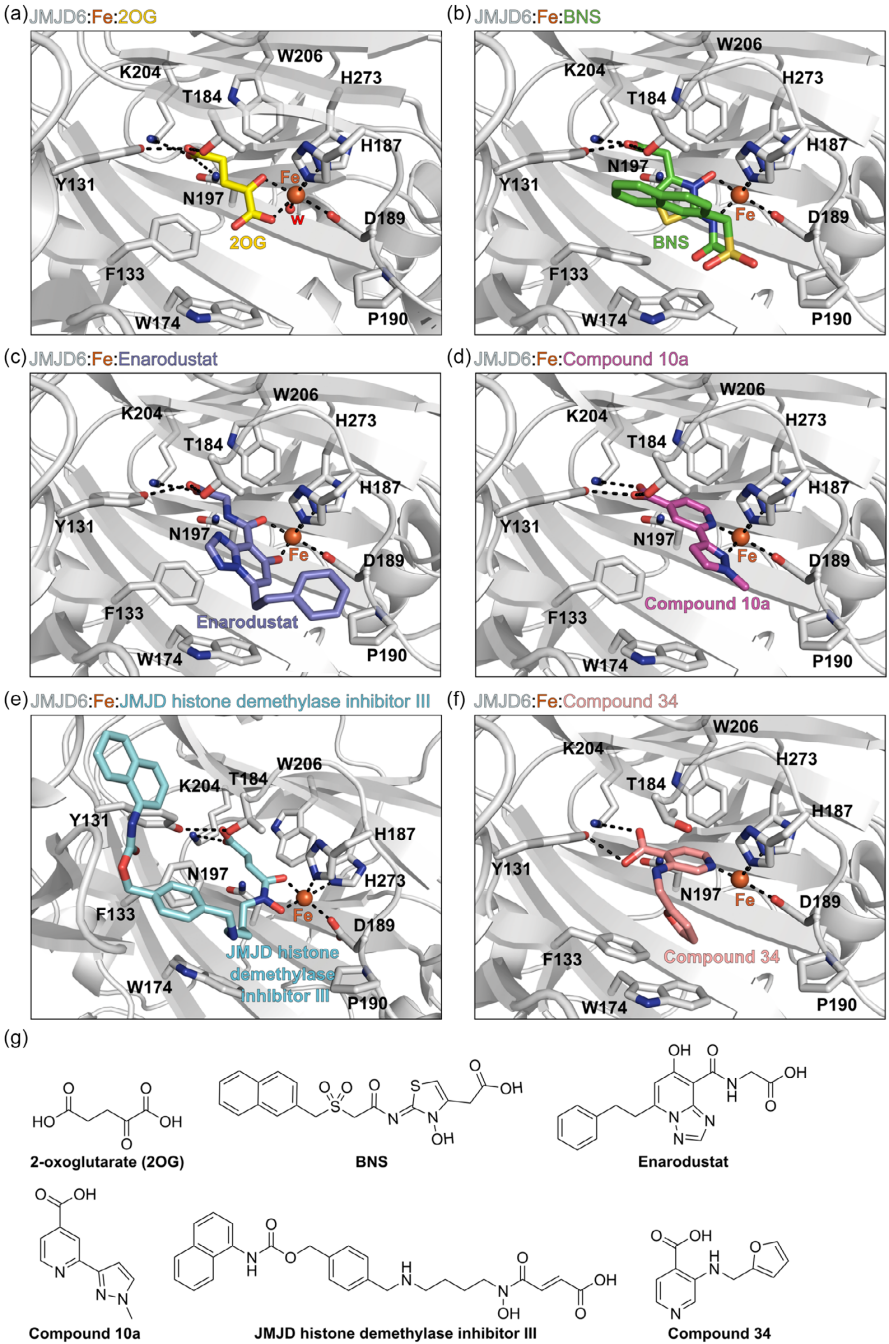
Docking studies predict that Compound 10a, BNS, Enarodustat, JMJD histone demethylase inhibitor III and Compound 34 likely bind to the active site of JMJD6 in a 2OG‐competing manner. a) View from a reported JMJD6:Fe:2OG structure (PDB ID: 6GDY; yellow: 2OG).^[^ ^4^ ^]^ b–f) Views from: b) the JMJD6:Fe:BNS docking prediction (green: BNS^[^ ^92^ ^,^ ^93^ ^]^), c) the JMJD6:Fe:Enarodustat docking prediction (violet: Enarodustat^[^ ^96^ ^]^), d) the JMJD6:Fe:Compound 10a docking prediction (pink: Compound 10a^[^ ^33^ ^]^), e) the JMJD6:Fe:JMJD histone demethylase inhibitor III docking prediction (cyan: JMJD histone demethylase inhibitor III^[^ ^82^ ^]^) and f) the JMJD6:Fe:Compound 34 docking prediction (salmon: compound 34^[^ ^84^ ^]^). Color code: light gray: JMJD6, orange: iron, red: oxygen, blue: nitrogen, gold: sulfur. w: water. Computational modeling was performed using GOLD 5.1^[^ ^104^ ^]^ as described in the Supporting Information. g) 2‐Oxoglutarate, BNS,^[^ ^92^, ^93^ Enarodustat,^[^ ^96^ ^]^ Compound 10a,^[^ ^33^ ^]^ JMJD histone demethylase inhibitor III,^[^ ^82^ ^]^ and Compound 34.^[^ ^84^ ^]^

The computational predictions indicate that all of the five modeled inhibitors can bind JMJD6 at its active site (**Figure** **2b–f**), as crystallographically observed for these and structurally related small molecules in complex with 2OG oxygenases.^[^ ^25^ ^,^ ^45^ ^,^ ^84^ ^,^ ^100^ ^,^ ^107^ ^]^ Compound 10a, BNS, Enarodustat, and JMJD histone demethylase inhibitor III are predicted to bind to Fe(II) in a bidentate manner similar to that observed for 2OG in a reported JMJD6:Fe:2OG structure (PDB ID:6GDY, Figure 2a).^[^ ^4^ ^]^ By contrast, Compound 34 is predicted to bind to Fe(II) through a monodentate interaction with its pyridine *N* atom (Figure 2f).

The 2OG C‐5 carboxylate group is crystallographically observed to interact with the side chains of the JMJD6 active site residues Y131, T184, N197, and K204 (Figure 2a).^[^ ^4^ ^]^ The carboxylate group of Compound 10a, BNS, Enarodustat, JMJD histone demethylase inhibitor III and Compound 34 is predicted to mimic the 2OG C‐5 carboxylate interactions with at least two of these residues (Figure 2b–f). Note that the T184 side chain is predicted to adopt a conformation in the modeled JMJD6:Fe:Compound 34 complex which differs from that in the reported JMJD6:Fe:2OG structure, likely to accommodate the C‐3 amino substituent of Compound 34.

The modeling predictions indicate that the F133 and/or Y174 side chains might form hydrophobic interactions with the naphthalene unit of BNS, the phenyl rings of Enarodustat and JMJD histone demethylase inhibitor III, and the furan ring of Compound 34, which likely contributes to their relatively high levels of inhibition potency (Figure 2). Although the JMJD6 active site may undergo conformational changes upon substrate/inhibitor binding, as frequently observed with 2OG oxygenases other than JMJD6,^[^ ^108^ ^–^ ^112^ ^]^ and the oligomeric nature of JMJD6 may affect inhibitor binding,^[^ ^7^ ^,^ ^113^ ^]^ the docking predictions indicate that there is sufficient space within the JMJD6 active site to allow for structural modification of the five inhibitors to optimize inhibition potency, selectivity, and physicochemical properties.

## 3 Conclusions

Although JMJD6 is a promising cancer target,^[^ ^20^ ^,^ ^22^ ^,^ ^32^ ^]^ relatively little is known about how its apparently multiple cellular activities relate to its biological roles, including in disease.^[^ ^4^ ^]^ Potent and selective small‐molecule JMJD6 inhibitors may enable cellular and in vivo functional assignment studies and investigations on the therapeutic potential of JMJD6 inhibition. JMJD6 inhibitor development, however, has likely been hindered by a lack of efficient assays that enable quantification of JMJD6 activity using isolated recombinant enzyme.

We developed robust SPE–MS assays suitable for inhibition studies (Figure S2, Supporting Information) that directly monitor the JMJD6‐catalyzed hydroxylation of BRD4_511–550_, which is derived from the reported cellular JMJD6 substrate BRD4.^[^ ^1^ ^,^ ^56^ ^]^ The JMJD6 and BRD4_511–550_ concentrations are reduced 200‐ and 100‐fold, respectively, compared with reported MALDI‐TOF MS‐ and LC–MS‐based assays,^[^ ^22^ ^,^ ^35^ ^]^ both reducing JMJD6/BRD4_511–550_ consumption and improving assay sensitivity.^[^ ^22^ ^,^ ^35^ ^]^ Importantly, the analysis of a 384‐well plate can be performed in ≈100 min (i.e., one well sampled every 15 s), taking advantage of the SPE–MS RapidFire setup, which represents a ≈20–40‐fold increase in throughput compared to the reported LC–MS assays,^[^ ^22^ ^,^ ^35^ ^]^ based on a 5–10 min run time per LCMS sample. The combined results reveal that the SPE–MS JMJD6 inhibition assays are of excellent robustness, as reflected by high Z’‐factors,^[^ ^114^ ^]^ signal‐to‐noise (S/N) ratios, and signal‐to‐background (S/B) ratios (Figure S2b–d, Supporting Information).

The SPE–MS inhibition assays enabled investigations on the effects of the reported JMJD6‐selective inhibitors WL12,^[^ ^32^ ^]^ SKLB325,^[^ ^20^ ^]^ and Compound 7p^[^ ^33^ ^]^ on isolated recombinant JMJD6 (Table 3, entries i–iii). Importantly, the results reveal that WL12^[^ ^32^ ^]^ did not inhibit JMJD6 (IC_50_ > 50 µM), while SKLB325^[^ ^20^ ^]^ and Compound 7p^[^ ^33^ ^]^ inhibited JMJD6 with only moderate potency (IC_50_ ≈ 6.7 and 4.2 µM, respectively), suggesting that the results of cellular studies with these small‐molecules likely do not directly relate to the JMJD6 lysine‐residue hydroxylation activity. By contrast, the carboxylic acid analogue of Compound 7p (i.e., Compound 10a^[^ ^33^ ^]^) was a highly efficient JMJD6 inhibitor (IC_50_ ≈ 0.07 µM; Table 3, entry iv). Compound 10a is thus an attractive scaffold for the development of improved JMJD6‐selective inhibitors, given its relatively high ligand efficiency (LE = 0.67)^[^ ^115^ ^]^ and that its synthesis is technically simple, high yielding, and can be modified to independently vary its pyridine and pyrazole groups (Scheme S1, Supporting Information).^[^ ^33^ ^]^

Several efficient JMJD6 inhibitors other than Compound 10a were identified which represent promising scaffolds for future JMJD6 inhibitor optimization (Table 4), in particular the clinically used PHD inhibitors Enarodustat^[^ ^96^ ^]^ (IC_50_ ≈ 1.2 µM) and Desidustat^[^ ^97^ ^]^ (IC_50_ ≈ 2.7 µM), but also TP0463518^[^ ^95^ ^]^ (IC_50_ ≈ 1.1 µM), Compound 34^[^ ^84^ ^]^ (IC_50_ ≈ 2.6 µM), the *N‐*hydroxythiazoles BNS^[^ ^92^ ^,^ ^93^ ^]^ and Compound 42^[^ ^45^ ^]^ (IC_50_ ≈ 0.16 and 0.37 µM, respectively), and the JMJD histone demethylase inhibitor III^[^ ^82^ ^]^ (IC_50_ ≈ 0.10 µM). Many of these scaffolds have been previously used in structure–activity relationship studies and reported derivatives may display improved potency and/or selectivity for JMJD6 inhibition.^[^ ^45^ ^,^ ^84^ ^,^ ^92^ ^,^ ^96^ ^,^ ^116^ ^]^ Although cellular and in vivo studies are required to validate the effects of these compounds on JMJD6 activity, the SPE–MS results indicate that JMJD6 inhibition may contribute to phenotypes reported following their use in cells.

The observation that both Enarodustat^[^ ^96^ ^]^ and Desidustat^[^ ^97^ ^]^ manifest relatively efficient inhibition of isolated JMJD6 is of interest given that they are approved for clinical use.^[^ ^98^ ^,^ ^117^ ^]^ The short‐ and long‐term effects of JMJD6 inhibition by Enarodustat^[^ ^96^ ^]^ and Desidustat^[^ ^97^ ^]^ should therefore be investigated, given that they are used over a relatively long time period to treat CKD‐associated anemia. As judged by IC_50_ comparison, Enarodustat and Desidustat inhibit JMJD6 approximately three‐ and twofold less efficiently than PHD2 (JMJD6 IC_50_s: 1.2 and 2.7 µM; PHD2 IC_50_s: 0.38 and 1.3 µM, respectively).^[^ ^100^ ^]^ Thus, the possibility of (off‐target) JMJD6 inhibition should be considered when evaluating cell‐based and in vivo studies with these and structurally related PHD inhibitors. It may be useful to test PHD inhibitors for inhibition of JMJD6 and 2OG oxygenase other than the PHDs and JMJD6, prior to entering clinical studies, due to the potentially severe phenotypes associated with JMJD6 inhibition.^[^ ^13^ ^,^ ^14^ ^,^ ^118^, ^119^ ^–^ ^120^ ^]^ Note that recent reports suggest that the clinical use of PHD inhibitors may be associated with side effects,^[^ ^121^ ^]^ perhaps reflecting inhibition of 2OG oxygenases other than the PHDs. The SPE–MS JMJD6 assay described here may help enable the development of more selective and safer 2OG oxygenase inhibitors suitable for clinical use.

## 4 Experimental Section

4.1

4.1.1

##### JMJD6 Production and Purification

Isolated recombinant full‐length human JMJD6 with a N‐terminal His_6_‐tag was produced in *Escherichia coli* BL21 (DE3) cells and purified as reported.^[^ ^1^ ^,^ ^56^ ^]^ Following Ni(II)‐affinity and size‐exclusion chromatography, His_6_‐JMJD6 was >95% pure by SDS‐PAGE and MS analysis. Purified His_6_‐JMJD6 was stored at −78 °C; fresh aliquots were used for all JMJD6 assays.

##### JMJD6 SPE–MS Inhibition Assay

Solutions of the small molecules (20 mM in DMSO) were dry dispensed across 384‐well polypropylene V‐bottom assay microplates (Greiner) in an approximately threefold and 11‐point dilution series (100 µM inhibitor top concentration; 0.5%_v/v_ final DMSO assay concentration) using an ECHO 550 acoustic dispenser (Labcyte). DMSO and 2,4‐PDCA (final assay concentration: 500 µM) were used as negative and positive inhibition controls, respectively. Each reaction was performed in technical duplicates in adjacent wells of the assay plates.

Cosubstrate/cofactor stock solutions (*L*‐ascorbic acid, LAA: 50 mM in Milli‐Q Ultrapure grade water; 2‐oxoglutarate, 2OG: 20 mM in Milli‐Q Ultrapure grade water; ammonium iron(II) sulfate hexahydrate, FAS, (NH_4_)_2_Fe(SO_4_)_2_·6H_2_O: 400 in 20 mM aqueous HCl diluted to 1 mM in Milli‐Q Ultrapure grade water) were freshly prepared from commercial solids (Sigma–Aldrich) on the day of the experiment. BRD4_511–550_ (LAELQEQLKAVHEQLAALSQPQQNKPKKKEKDKKEKKKEK) was synthesized by solid‐phase peptide synthesis and was purified by GL Biochem (Shanghai) Ltd (Shanghai, China). Stock solutions of BRD4_511–550_ (10 mM in water) were stored at −20 °C.

The ice‐cold enzyme mixture, containing 0.1 µM His_6_‐JMJD6 (i.e., 2 × final concentration) in Tris buffer (50 mM; pH 7.5), was dispensed across the inhibitor‐containing 384‐well plates (25 µL per well) using a multidrop dispenser (ThermoFischer Scientific) at room temperature under an ambient atmosphere. The plates were centrifuged (1000 rpm, 5 s) and incubated for 15 min at ambient temperature. The substrate mixture (2 × final concentration), containing BRD4_511–550_ (2 µM), LAA (200 µM), 2OG (200 µM), and FAS (4 µM) in Tris buffer (50 mM; pH 7.5), was then dispensed across the plates (25 µL per well) using the multidrop dispenser. The plates were centrifuged (1000 rpm, 5 s) and incubated for 20 min at ambient temperature. The enzyme reaction was then stopped by the addition of 10%_v/v_ aqueous formic acid (5 μL per well) using a 16‐channel electronic pipette (ThermoFischer Scientific). The plates were then centrifuged (1000 rpm, 30 s) and analyzed by MS.

MS analyses were performed using a RapidFire RF 365 high‐throughput sampling robot (Agilent) attached to an iFunnel Agilent 6550 accurate mass quadrupole time‐of‐flight (Q‐TOF) mass spectrometer, which was operated in the positive ionization mode. Assay samples were aspirated under vacuum for 0.4 s and then loaded onto a C4 SPE cartridge. After loading, the C4 SPE cartridge was washed with 0.1%_v/v_ aqueous formic acid to remove nonvolatile buffer salts (5 s, 1.5 mL min^−1^). BRD4_511–550_ and the corresponding hydroxylated product peptides were eluted from the SPE cartridge with 0.1%_v/v_ aqueous formic acid in 75/25_v/v_ acetonitrile/water into the mass spectrometer (5 s, 1.6 mL min^−1^); the SPE cartridge was subsequently re‐equilibrated with 0.1%_v/v_ aqueous formic acid (1 s, 1.25 mL min^−1^).

Peaks corresponding to the *m/z* + 5 charge states of BRD4_511–550_ and the corresponding mono‐ and dihydroxylated product peptides were extracted from the ion chromatogram and integrated using RapidFire Integrator 4.3.0 (Agilent). Peak area data were exported into Microsoft Excel and used to calculate the JMJD6 activity (%) using the following equation:

(1) $$\mathrm{JMJD}6\;\mathrm{activity}\;(\%)=100\times (\mathrm{integral}\;\mathrm{monohydroxylated}\;\mathrm{peptide}+2\times \mathrm{integral}\;\mathrm{dihydroxylated}\;\mathrm{peptide})/(\mathrm{integral}\;\mathrm{BRD}4_{511‐550}+\mathrm{integral}\;\mathrm{monohydroxylated}\;\mathrm{peptide}+\mathrm{integral}\;\mathrm{dihydroxylated}\;\mathrm{peptide})$$

From the raw data, dose–response curves (normalized to the 2,4‐PDCA and DMSO controls) were obtained by nonlinear regression (GraphPad Prism 5), which were used to determine IC_50_ values.

## Conflict of Interest

The authors declare no conflict of interest.

## Supporting information

Supplementary Material

## Data Availability

The data that support the findings of this study are available in the *supplementary material* of this article.
