# Supplementary material for: Profiling Inhibitor Scaffolds for the Cancer Target Jumonji‐C Domain‐Containing Protein 6
Source: ChemMedChem. 2025 Oct 31;20(23):e202500682. doi: 10.1002/cmdc.202500682 (PMC12677844; doi:10.1002/cmdc.202500682)
Supplement: Supplementary file 1 — Supplementary Material [file CMDC-20-e202500682-s001.pdf]

Supporting Information

Profiling Inhibitor Scaffolds for the Cancer Target Jumonji-C Domain-Containing Protein 6

Thomas P. Corner[1,+,#], Eidarus Salah[1,+], Anthony Tumber[1,+], James P. Holt-Martyn[1], Lennart Brewitz[1,*], and Christopher J. Schofield[1,*]

[1]Chemistry Research Laboratory, Department of Chemistry and the Ineos Oxford Institute for Antimicrobial Research, University of Oxford, 12 Mansfield Road, Oxford OX1 3TA, United Kingdom.

*Email: lennart.brewitz@chem.ox.ac.uk or christopher.schofield@chem.ox.ac.uk

[+]These authors contributed equally to this work.

[#]Present Address: Department of Molecular, Cellular, and Developmental Biology, Yale University, New Haven, CT 06511, United States of America.

Table of contents

1. Supporting figures....S3
2. Supporting synthetic schemes....S7
3. Computational methods....S9
4. General synthesis information....S10
5. Synthetic procedures and compound characterisations....S11
6. ^{1}H and ^{13}C NMR spectra of compounds prepared for this study....S14
7. References....S20

1. Supporting figures

Supporting Figure S1. Mass spectrometric analysis of JMJD6-catalysed BRD4$_{511\text{-}550}$ hydroxylation.

Reactions were performed to investigate the time-dependent hydroxylation of BRD4$_{511\text{-}550}$ catalysed by isolated recombinant full-length His$_6$-JMJD6. His$_6$-JMJD6 (final concentration: 50 nM) was incubated in the presence of 2OG (100 µM), $(NH_4)_2Fe(SO_4)_2 \cdot 6H_2O$ (2 µM), BRD4$_{511\text{-}550}$ (1 µM) and *L*-ascorbic acid (100 µM) in Tris buffer (50 mM, pH 7.5, 20 ˚C) in Greiner 384-well polypropylene assay plates (reaction volume: 50 µL). Reactions were stopped at the specified times by addition of 10%$_{v/v}$ aqueous formic acid (5 µL). The extent of BRD4$_{511\text{-}550}$ mono- and di-hydroxylation was determined by solid-phase extraction coupled to mass spectrometry (SPE-MS) using a C4 SPE cartridge. Data are means of three independent runs (n = 3; mean ± standard deviation, SD). The results reveal that JMJD6 catalysed ~30% mono-hydroxylation and <5% di-hydroxylation of BRD4$_{511\text{-}550}$ after 20 min, while at longer incubation times, levels of the di-hydroxylated product were increased. Thus, stopping the JMJD6 reaction after 20 min incubation was considered to be preferred for robust inhibition assays.

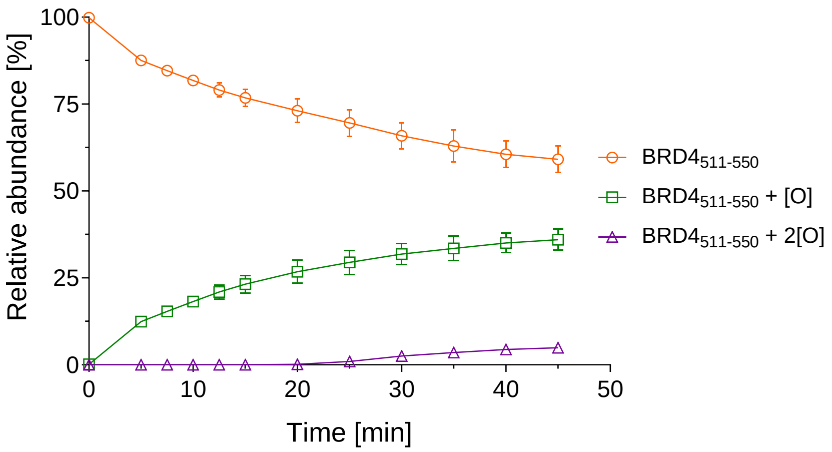

Supporting Figure S2. Validation of the JMJD6 SPE-MS inhibition assay robustness.

(**a**) Dose-response curves for the broad-spectrum 2OG oxygenase inhibitors: 2,4-PDCA (purple circles), *N*-oxalylglycine (NOG; orange squares) and IOX1 (green triangles). The dose-response curves are means of technical duplicates (n = 2; mean ± SD). The mean of independent duplicates each composed of technical duplicates was used to determine IC_{50} values (shown in **Table 2**). (**b**) Z'-factors[1] (purple circles), (**c**) signal-to-noise (S/N) ratios (orange squares), and (**d**) signal-to-background (S/B) ratios (green triangles) for the JMJD6 inhibition assay plates analysed in this work. Z'-factors >0.5 indicate a stable and robust assay of high quality.[1]

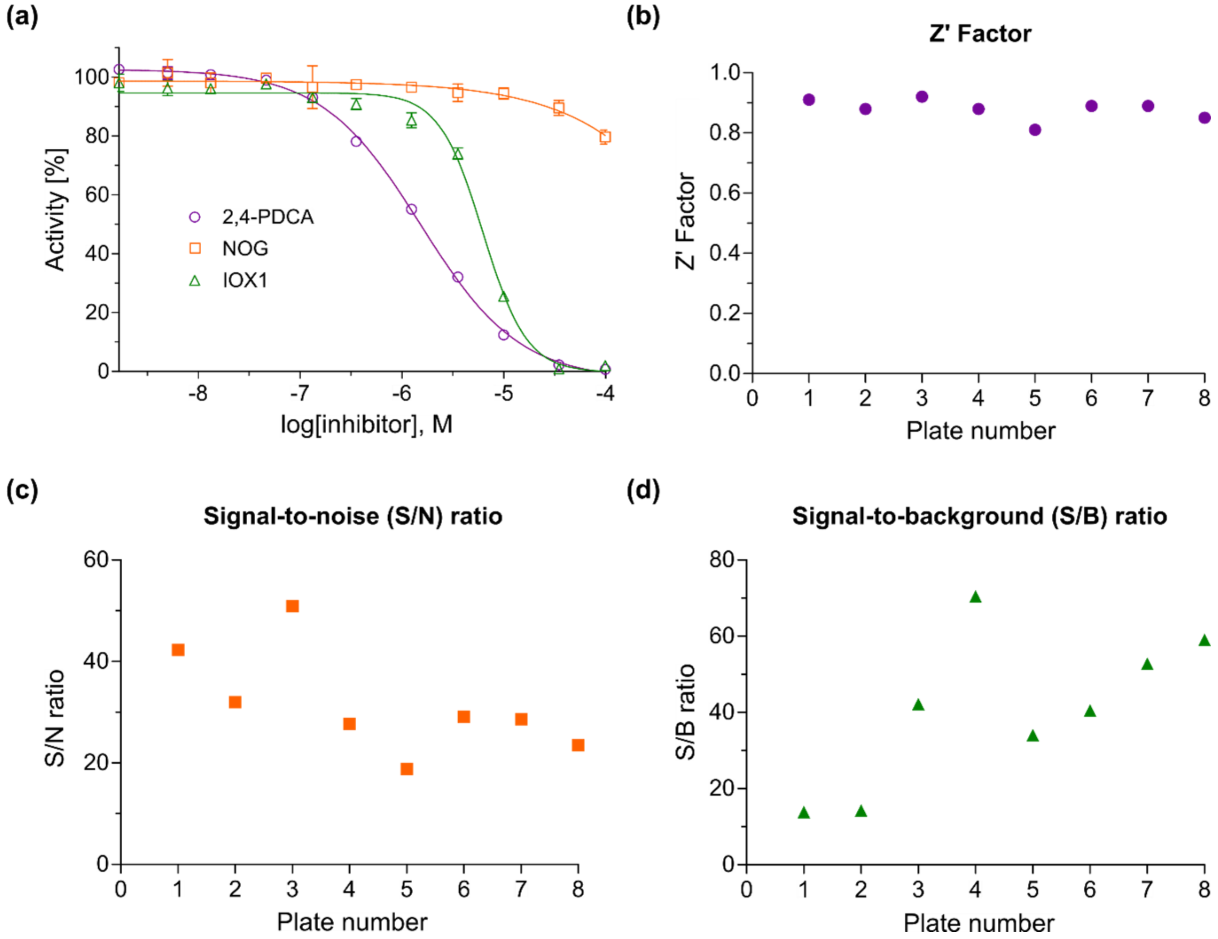

Supporting Figure S3. Effect of DMSO on JMJD6 catalysis.

Reactions were performed to investigate the effect of DMSO on the JMJD6-catalysed $BRD4_{511\text{-}550}$ hydroxylation reaction. His_6-JMJD6 (final concentration: 50 nM) was incubated in the presence of varied concentrations of DMSO (0 – $2\%_{v/v}$, as specified), 2OG (25 µM), $(NH_4)_2Fe(SO_4)_2 \cdot 6H_2O$ (2 µM), $BRD4_{511\text{-}550}$ (2 µM) and *L*-ascorbic acid (100 µM) in Tris buffer (50 mM, pH 7.5, 20 ˚C) in Greiner 96-deep well polypropylene assay plates (reaction volume: 500 µL). The extent of $BRD4_{511\text{-}550}$ hydroxylation was monitored by SPE-MS using a C4 SPE cartridge. Data are means of three independent runs (n = 3; mean ± SD). The results reveal that the presence of DMSO did not affect JMJD6 in the tested concentration range.

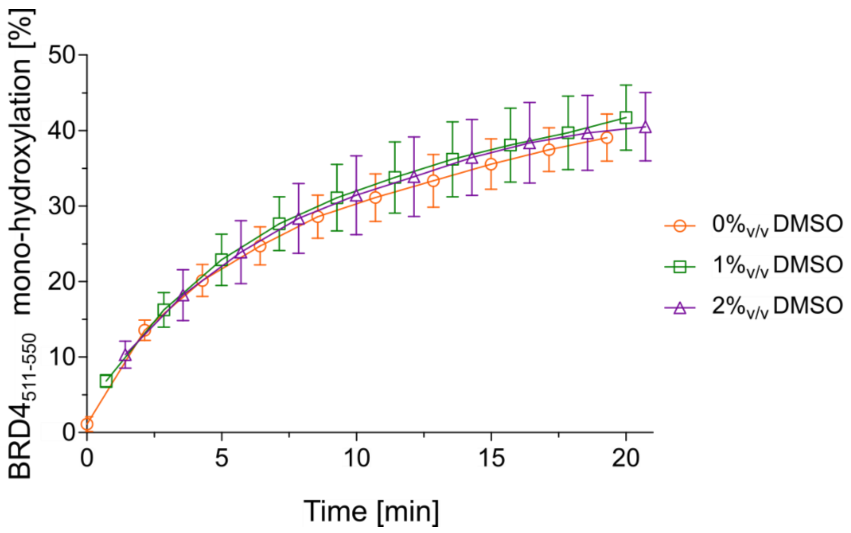

Supporting Figure S4. Representative dose-response curves of small-molecule JMJD6 inhibitors.

SPE-MS inhibition assays were performed as described in the Experimental Section using isolated recombinant His_6-JMJD6 (50 nM), 2OG (100 µM), $(NH_4)_2Fe(SO_4)_2 \cdot 6H_2O$ (2 µM), $BRD4_{511-550}$ (1 µM) and *L*-ascorbic acid (100 µM) in Tris buffer (50 mM, pH 7.5) in Greiner 384-well polypropylene assay plates (reaction volume: 50 µL). Dose-response curves are means of technical duplicates (n = 2; mean ± SD). Means of independent duplicates each composed of technical duplicates were used to determine IC_{50} values.

(**a**) **2,4-PDCA**[2]: pink circles, **IOX1**[3]: green squares, **Daminozide**[4]: blue triangles, **WL12**[5]: purple inverse triangles, **SKLB325**[6]: orange diamonds, **Compound 7p**[7]: blue hexagons, **Compound 10a**[7]: beige half-filled circles.

(**b**) **JMJD histone demethylase inhibitor III**[8]: pink circles, **ML324**[9]: green squares, **Compound 34**[10]: blue triangles, **QC6352**[11]: purple inverse triangles, **TACH101**[12]: orange diamonds, **AS-8351**[13]: blue hexagons.

(**c**) **Compound 42**[14]: pink circles, **BNS**[15]: green squares, **TP0463518**[16]: blue triangles, **Enarodustat**[17]: purple inverse triangles, **Desidustat**[18]: orange diamonds, **Compound 48**[19]: blue hexagons.

(**d**) **IOX2**[20]: pink circles, **Daprodustat**[21]: green squares, **Vadadustat**[22]: blue triangles, **Molidustat**[23]: purple inverse triangles, **MK-8617**[24]: orange diamonds.

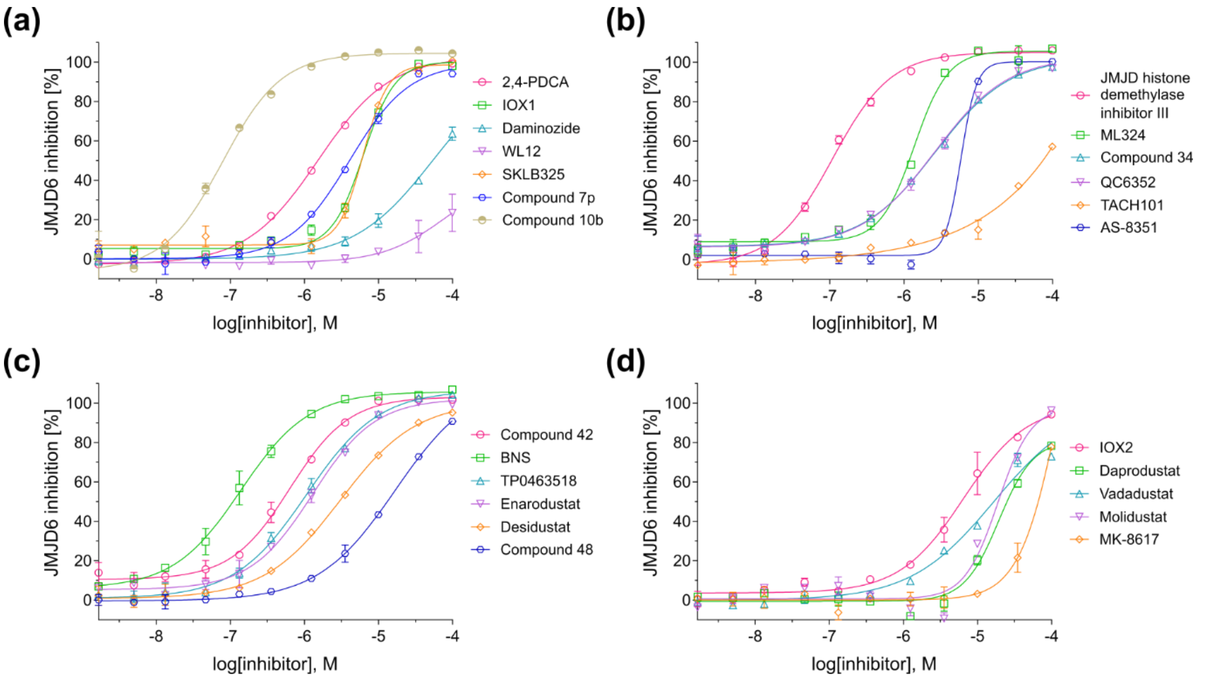

2. Supporting synthetic schemes

Supporting Scheme S1. Synthesis of the reported JMJD6 inhibitors Compound 7p and Compound 10b.

The reported JMJD6 inhibitor Compound 7p[7] was synthesized in four steps from commercial 2-bromoisonicotinic acid **1** (5% overall yield). 4-(*N*,*N*-Dimethyl)aminopyridine-mediated esterification of **1** using Boc_2O gave *tert*-butyl ester **2**, which was then coupled with commercial 1-methyl-3-(4,4,5,5-tetramethyl-1,3,2-dioxaborolan-2-yl)-1*H*-pyrazole using a Pd-catalyzed Suzuki coupling reaction[25] to yield **3**. *tert*-Butyl ester cleavage with TFA generated the reported JMJD6 inhibitor Compound 10b,[7] which was then coupled with 2,2,2-trifluoroethanol in the presence of EDC·HCl to afford the reported JMJD6 inhibitor Compound 7p.[7]

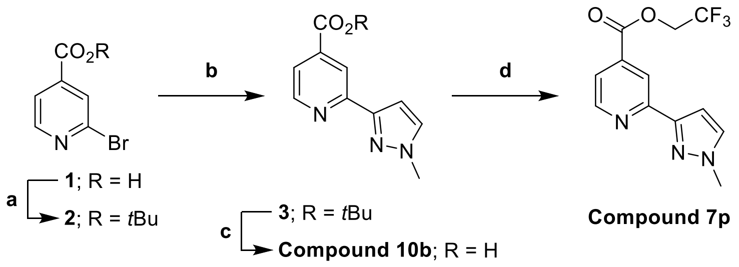

Reagents and conditions: (**a**) Boc_2O, 4-(*N*,*N*-dimethyl)aminopyridine, THF, 0 °C to rt, 84%; (**b**) 1-methyl-3-(4,4,5,5-tetramethyl-1,3,2-dioxaborolan-2-yl)-1*H*-pyrazole, Pd(dppf)Cl_2, K_3PO_4, 1,4-dioxane/H_2O, 120 °C (sealed tube), 60%, (**c**) TFA, CH_2Cl_2, 0 °C to rt, 43%; (**d**) 2,2,2-trifluoroethanol, EDC·HCl, 4-(*N*,*N*-dimethyl)aminopyridine, $^iPr_2NEt$, CH_2Cl_2, 0 °C to rt, 23%.

Supporting Scheme S2. Synthesis of the reported KDM4/5 inhibitor Compound 34.

The reported KDM4/5 inhibitor Compound 34[10] was synthesised in two steps from commercial methyl 3-bromoisonicotinate **4** (66% overall yield). First, **4** was coupled with furan-2-ylmethanamine using a Pd-catalysed Buchwald-Hartwig reaction[26] to afford methyl ester **5**. Lithium hydroxide-mediated saponification of **5** generated the reported KDM4/5 inhibitor Compound 34.

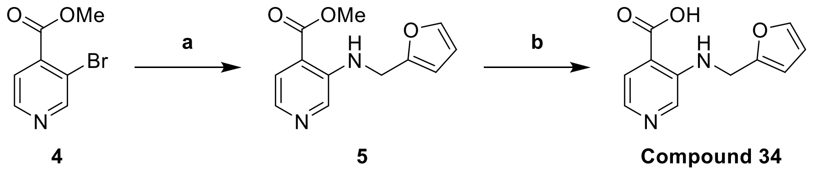

Reagents and conditions: (**a**) furan-2-ylmethanamine, $Pd(OAc)_2$, Xantphos[27], Cs_2CO_3, toluene, 150 ˚C (sealed tube), 86%; (**b**) LiOH, $MeOH/H_2O$, 0 °C to rt, 77%.

3. Computational methods

Protein-ligand docking

A reported JMJD6:Fe:2OG (PDB ID: 6GDY[28]) complex structure was downloaded from the Protein Data Bank (https://www.rcsb.org/):[29] Hydrogen atoms were added, and Asn/Gln/His residues were checked for conformational isomers/flips with REDUCE,[30] using the MolProbity server.[31] The pKa values of ionizable groups were calculated using PropKa[32] and ionizable groups were protonated using Pymol (version 4.6.0)[33] at pH 7.5. Alternative side chain conformations, bound ligands, and all crystallographic waters were removed using Pymol. Docking studies were performed using Gold software (version 5.1).[34] The side chains conformations of Y131, F133 and T184 (for Compound 34 only) were set as flexible; the side chain conformations of all other active site residues were kept rigid. For each ligand, 25 genetic algorithm (GA) runs were carried out; the GoldScore-CS consensus scoring function of Gold was used to evaluate the predicted ligand binding poses.[35] The binding site was defined as all atoms within 20 Å of the catalytic Fe(II). The 'Detect internal H bonds' and 'Flip amide bond' ligand flexibility parameters were enabled. The 'Allow early termination' option was disabled. The coordination geometry of the Fe(II) ion was set as octahedral. All other settings were used as the defaults.

4. General synthesis information

All reagents were purchased from commercial sources (Sigma-Aldrich, Inc.; Fluorochem Ltd; Tokyo Chemical Industries) and used as received. Compound 42,[14] IOX5[36] and Compound 48[19] were synthesised following reported procedures.

Anhydrous solvents (Sigma-Aldrich, Inc.) were kept under an atmosphere of nitrogen. Purifications were performed using Biotage Isolera One or Biotage Selekt purification machines (wavelengths monitored: 254 and 280 nm) equipped with pre-packed Biotage® Sfär Duo flash chromatography cartridges. The cartridge type and size, as well as solvent gradients (in column volumes, CV) used, are specified in the individual experimental procedures. HPLC grade solvents (Sigma-Aldrich, Inc.) were used for purifications, reaction work-ups, and extractions.

Thin layer chromatography (TLC) was carried out using Merck silica gel 60 F_{254} TLC plates and visualized using UV light. Melting points (m.p.) were determined using a Stuart SMP-40 automated melting point apparatus. Infrared (IR) spectroscopy was performed using a Bruker Tensor-27 Fourier transform infrared (FT-IR) spectrometer. High-resolution mass spectrometry (HRMS) was performed using electrospray ionization (ESI) mass spectrometry (MS) in the positive or negative ionization mode employing a Thermo Scientific Exactive mass spectrometer (ThermoFisher Scientific); data are presented as a mass-to-charge ratio (*m/z*).

Nuclear magnetic resonance (NMR) spectroscopy was performed using a Bruker AVANCE AVIII HD 600 MHz machine equipped with a 14.1 T magnet and with a 5 mm BB-F/^{1}H Prodigy N_2 cryoprobe or a Bruker Avance III HD 500 MHz machine equipped with a 11.75 T magnet. Chemical shifts for ^{1}H NMR are reported in parts per million (ppm) downfield from tetramethylsilane and are referenced to the residual protium in the NMR solvent ($CDCl_3$: δ = 7.26 ppm; DMSO-d_6: δ = 2.50 ppm). For ^{13}C NMR, chemical shifts are reported in the scale relative to the NMR solvent ($CDCl_3$: δ = 77.2 ppm; DMSO-d_6: δ = 39.5 ppm). For ^{19}F NMR, chemical shifts are reported in the scale relative to $CFCl_3$. NMR data are reported as follows: chemical shift, multiplicity (s: singlet, d: doublet, dd: doublet of doublets, t: triplet, q: quartet, m: multiplet, br: broad signal), coupling constant (*J*, Hz; accurate to 0.5 Hz), and integration. All compounds were >95% pure by ^{1}H and ^{13}C NMR analyses. ^{1}H and ^{13}C NMR spectra of compounds prepared for this study are shown in Section 6 of the Supporting Information.

5. Synthetic procedures and compound characterisations

tert-Butyl 2-bromoisonicotinate (2)

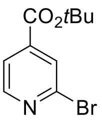

To a solution of 2-bromoisonicotinic acid **1** (1.01 g, 5.0 mmol, 1.0 equiv.) in anhydrous tetrahydrofuran (20 mL) were added 4-(*N*,*N*-dimethylamino)pyridine (611 mg, 5.0 mmol, 1.0 equiv.) and di-*tert*-butyldicarbonate (1.31 g, 6.0 mmol, 1.2 equiv.) under a N_2 atmosphere at 0 °C. The reaction mixture was allowed to warm to ambient temperature and stirred for 14 h; the solvent was then removed under reduced pressure. The residue was dissolved in ethyl acetate and washed with H_2O, then brine. The organic layer was dried over anhydrous Na_2SO_4, filtered, concentrated under reduced pressure, and purified by column chromatography (50 g Sfär Silica D; 60 mL/min; 100%$_{v/v}$ cyclohexane (2 CV), followed by a linear gradient (14 CV): 0%$_{v/v}$ → 10%$_{v/v}$ ethyl acetate in cyclohexane) to afford ester **2** (1.09 g, 4.2 mmol, 84%).

Colorless oil; ^{1}H NMR (500 MHz, 300 K, $CDCl_3$): δ = 8.48 – 8.43 (m, 1H), 7.93 (s, 1H), 7.74 – 7.71 (m, 1H), 1.57 ppm (s, 9H); ^{13}C NMR (126 MHz, 300 K, $CDCl_3$): δ = 162.7, 150.8, 142.8, 141.9, 127.8, 122.0, 83.3, 28.1 ppm; IR (film): $\tilde{\nu}$ = 3062, 2981, 1725, 1550, 1365, 1305, 1168, 1143 cm^{-1}: HRMS (ESI): *m/z* calcd for $C_{10}H_{13}BrNO_2$ [*M*+H]$^+$: 258.0124, found: 258.0124.

tert-Butyl 2-(1-methyl-1*H*-pyrazol-3-yl)isonicotinate (3)

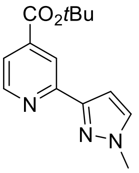

To a mixture of ester **2** (432 mg, 2.0 mmol, 1.0 equiv.), 1-methyl-3-(4,4,5,5-tetramethyl-1,3,2-dioxaborolan-2-yl)-1*H*-pyrazole (416 mg, 2.0 mmol, 1.0 equiv.), tripotassium phosphate (848 mg, 4.0 mmol, 2.0 equiv.) and [1,1'-bis(diphenylphosphino)ferrocene]palladium(II) dichloride (163 mg, 0.2 mmol, 0.1 equiv.) in a sealed 20 mL microwave reaction vial were added anhydrous dioxane (8 mL) and Milli-Q grade H_2O (2 mL) at ambient temperature. N_2 gas was bubbled through the reaction mixture for 15 min and the sealed reaction vessel was then heated in a 150 °C sand bath for 14 h, before being cooled to ambient temperature. The reaction mixture was then filtered through celite and diluted with ethyl acetate. The biphasic mixture was separated, and the organic layer was washed with H_2O, then brine. The organic layer was dried over anhydrous Na_2SO_4, filtered, concentrated under reduced pressure, and purified by column chromatography (25 g Sfär Silica D; 60 mL/min; 100%$_{v/v}$ cyclohexane (2 CV), followed by a linear gradient (14 CV): 0%$_{v/v}$ → 30%$_{v/v}$ ethyl acetate in cyclohexane) to afford pyrazole **3** (312 mg, 1.2 mmol, 60%).

Brown oil; ^{1}H NMR (600 MHz, 300 K, $CDCl_3$): δ = 8.72 (d, *J* = 5.0 Hz, 1H), 8.35 (s, 1H), 7.68 (d, *J* = 5.0 Hz, 1H), 7.41 (d, *J* = 2.0 Hz, 1H), 6.92 – 6.89 (m, 1H), 3.98 (s, 3H), 1.60 ppm (s, 9H); ^{13}C NMR (151 MHz, 300 K, $CDCl_3$): δ = 164.3, 153.1, 150.9, 149.8, 140.3, 131.8, 121.4, 119.4, 105.0, 82.5, 39.4, 28.2 ppm; IR (film): $\tilde{\nu}$ = 2981, 1721, 1604, 1352, 1300, 1168, 1103 cm^{-1}; HRMS (ESI): *m/z* calcd for $C_{14}H_{18}N_3O_2$ [*M*+H]$^+$: 260.1394, found: 260.1395.

Compound 10b[7]

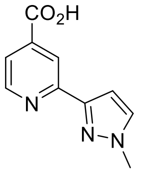

To a solution of pyrazole **3** (230 mg, 0.89 mmol, 1.0 equiv.) in anhydrous dichloromethane (1.4 mL) was added dropwise trifluoroacetic acid (0.68 mL, 8.9 mmol, 10 equiv.) at 0 °C under a N_2 atmosphere. The reaction mixture was allowed to warm to ambient temperature and stirred for 2 h, before being concentrated under reduced pressure. The residue was purified by reverse-phase column chromatography (30 g Sfär C18 Duo; 25 mL/min; 100% water (+ 0.1%$_{v/v}$ formic acid) (4 CV), followed by a linear gradient (20 CV): 0%$_{v/v}$ →30%$_{v/v}$ acetonitrile (+ 0.1%$_{v/v}$ formic acid) in water (+ 0.1%$_{v/v}$ formic acid)) to afford Compound 10b (78 mg, 0.38 mmol, 43%). The analytical data for Compound 10b are consistent with those reported.[7]

White solid, m.p.: >250 °C; ^{1}H NMR (500 MHz, 300 K, DMSO-*d*$_6$): δ = 13.69 (s, 1H), 8.74 (d, *J* = 5.0 Hz, 1H), 8.33 (s, 1H), 7.80 (d, *J* = 2.0 Hz, 1H), 7.69 (d, *J* = 5.0 Hz, 1H), 6.84 (d, *J* = 2.0 Hz, 1H), 3.94 ppm (s, 3H); ^{13}C NMR (126 MHz, 300 K, DMSO-*d*$_6$): δ = 166.2, 152.9, 150.3, 150.1, 138.8, 132.7, 121.1, 118.0, 104.1, 38.9 ppm; IR (film): $\tilde{\nu}$ = 3109, 1703, 1618, 1559, 1350, 1212, 1086 cm^{-1}; HRMS (ESI): *m/z* calcd for $C_{10}H_{10}N_3O_2$ [*M*+H]$^+$: 204.0768, found: 204.0768.

Compound 7p[7]

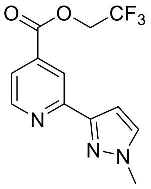

To a mixture of Compound 10b[7] (50 mg, 0.23 mmol, 1.0 equiv.), 4-(*N*,*N*-dimethylamino)pyridine (34 mg, 0.28 mmol, 1.2 equiv.) and 1-ethyl-3-(3-dimethylaminopropyl)carbodiimide hydrochloride (54 mg, 0.28 mmol, 1.2 equiv.) in anhydrous dichloromethane (1 mL) were added redistilled *N*,*N*-diisopropylethylamine (80 µL, 0.46 mmol, 2.0 equiv.) and 2,2,2-trifluoroethanol (17 µL, 0.25 mmol, 1.1 equiv.) under a N_2 atmosphere at 0 °C. The reaction mixture was allowed to warm to ambient temperature and stirred for 72 h, before being concentrated under reduced pressure. The crude residue was purified by reverse-phase column chromatography (12 g Sfär C18 Duo; 12 mL/min; 100% water (+ 0.1%$_{v/v}$ formic acid) (4 CV), followed by a linear gradient (20 CV): 0%$_{v/v}$ → 30%$_{v/v}$ acetonitrile (+ 0.1%$_{v/v}$ formic acid) in water (+ 0.1%$_{v/v}$ formic acid)) to afford Compound 7p (15 mg, 0.05 mmol, 23%). The analytical data for Compound 7p are consistent with those reported.[7]

White solid, m.p.: 102–103 °C; ^{1}H NMR (600 MHz, 300 K, DMSO-*d*$_6$): δ = 8.82 (d, *J* = 5.0 Hz, 1H), 8.34 (s, 1H), 7.83 (d, *J* = 2.0 Hz, 1H), 7.75 (d, *J* = 5.0 Hz, 1H), 6.86 (d, *J* = 2.0 Hz, 1H), 5.08 (q, *J* = 9.0 Hz, 2H), 3.95 ppm (s, 3H); ^{13}C NMR (151 MHz, 300 K, DMSO-*d*$_6$): δ = 163.3, 153.2, 150.8, 149.8, 136.1, 132.9, 123.5 (q, *J* = 277.5 Hz), 121.0, 117.7, 104.3, 61.0 (q, *J* = 35.5 Hz), 39.0 ppm; ^{19}F NMR (565 MHz, 300 K, DMSO-*d*$_6$): δ = –72.2 ppm (t, *J* = 9.0 Hz); IR (film): $\tilde{\nu}$ = 2961, 1746, 1608, 1350, 1224, 1165, 1042 cm^{-1}; HRMS (ESI): *m/z* calcd for $C_{12}H_{11}F_3N_3O_2$ [*M*+H]$^+$: 286.0798, found: 286.0791.

Methyl 3-((furan-2-ylmethyl)amino)isonicotinate (5)

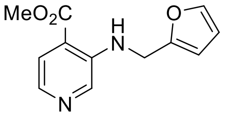

To a mixture of cesium carbonate (912 mg, 2.8 mmol, 1.4 equiv.), palladium acetate (18 mg, 0.08 mmol, 0.04 equiv.) and xantphos[27] (69 mg, 0.12 mmol, 0.06 equiv.) in a sealed 20 mL microwave reaction vial were sequentially added anhydrous toluene (8 mL), methyl 3-bromoisonicotinate **4** (432 mg, 2.0 mmol, 1.0 equiv.) and furan-2-ylmethanamine (0.21 mL, 2.4 mmol, 1.5 equiv.) at ambient temperature. N_2 gas was bubbled through the reaction mixture for 15 min and the sealed reaction vessel was then heated in a 150 °C sand bath for 14 h, before being cooled to ambient temperature. Then, the reaction mixture was concentrated under reduced pressure and purified by column chromatography (25 g Ultra; 50 mL/min; 100%$_{v/v}$ cyclohexane (3 CV), followed by a linear gradient (15 CV): 0%$_{v/v}$ → 25%$_{v/v}$ ethyl acetate in cyclohexane) to afford ester **5** (397 mg, 1.7 mmol, 86%).

Yellow solid, m.p.: 68–70 °C; ^{1}H NMR (600 MHz, 300 K, $CDCl_3$): δ = 8.32 (s, 1H), 7.94 (d, *J* = 5.0 Hz, 1H), 7.74 (s, 1H), 7.65 – 7.60 (m, 1H), 7.36 (dd, *J* = 2.0, 1.0 Hz, 1H), 6.31 (dd, *J* = 3.0, 2.0 Hz, 1H), 6.28 – 6.25 (m, 1H), 4.49 (d, *J* = 5.5 Hz, 2H), 3.88 ppm (s, 3H); ^{13}C NMR (151 MHz, 300 K, $CDCl_3$): δ = 167.9, 151.4, 144.5, 142.4, 136.5, 135.8, 123.3, 115.8, 110.5, 107.5, 52.2, 40.1 ppm; IR (film): $\tilde{\nu}$ = 3363, 2948, 1693, 1567, 1438, 1329, 1303, 1220, 1169 cm^{-1}: HRMS (ESI): *m/z* calculated for $C_{12}H_{13}N_2O_3$ [*M*+H]$^+$: 233.0926, found: 233.0921.

Compound 34[10]

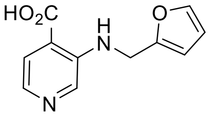

To a solution of ester **5** (116 mg, 0.50 mmol, 1.0 equiv.) in methanol (2.5 mL; HPLC grade) was added an aqueous solution of lithium hydroxide (0.4 M; 3.1 mL, 1.3 mmol, 2.5 equiv.) under an ambient atmosphere at 0 °C. The reaction mixture was allowed to slowly warm to ambient temperature, then stirred for 14 h; the methanol was then removed under reduced pressure. The solution was extracted three time with dichloromethane (the organic extracts were discarded); the aqueous layer was then acidified (pH~7) by the portionwise addition of Dowex® 50XW8 (H$^+$-form, mesh 200-400), filtered, and lyophilised to afford purified Compound 34 (84 mg, 0.38 mmol, 77%). The analytical data for Compound 34 are consistent with those reported.[10]

Orange solid, m.p.: >250 °C (decomposition); ^{1}H NMR (600 MHz, 300 K, D_2O): δ = 8.16 (s, 1H), 7.87 (d, *J* = 5.0 Hz, 1H), 7.54 (d, *J* = 5.0 Hz, 1H), 7.43 (d, *J* = 2.0 Hz, 1H), 6.38 – 6.36 (m, 1H), 6.33 – 6.30 (m, 1H), 4.45 ppm (s, 2H); ^{13}C NMR (151 MHz, 300 K, D_2O): δ = 173.7, 152.2, 143.5, 142.5, 137.3, 135.0, 127.6, 124.2, 110.4, 107.2, 39.9 ppm; IR (film): $\tilde{\nu}$ = 3316, 1616, 1575, 1504, 1434, 1384, 1247 cm^{-1}: HRMS (ESI): *m/z* calculated for $C_{11}H_{11}N_2O_3$ [*M*+H]$^+$: 219.0770, found: 219.0764.

6. ^{1}H and ^{13}C NMR spectra of compounds prepared for this study

tert-Butyl 2-bromoisonicotinate (2)

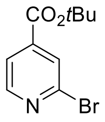

^{1}H NMR (500 MHz, 300 K, $CDCl_3$):

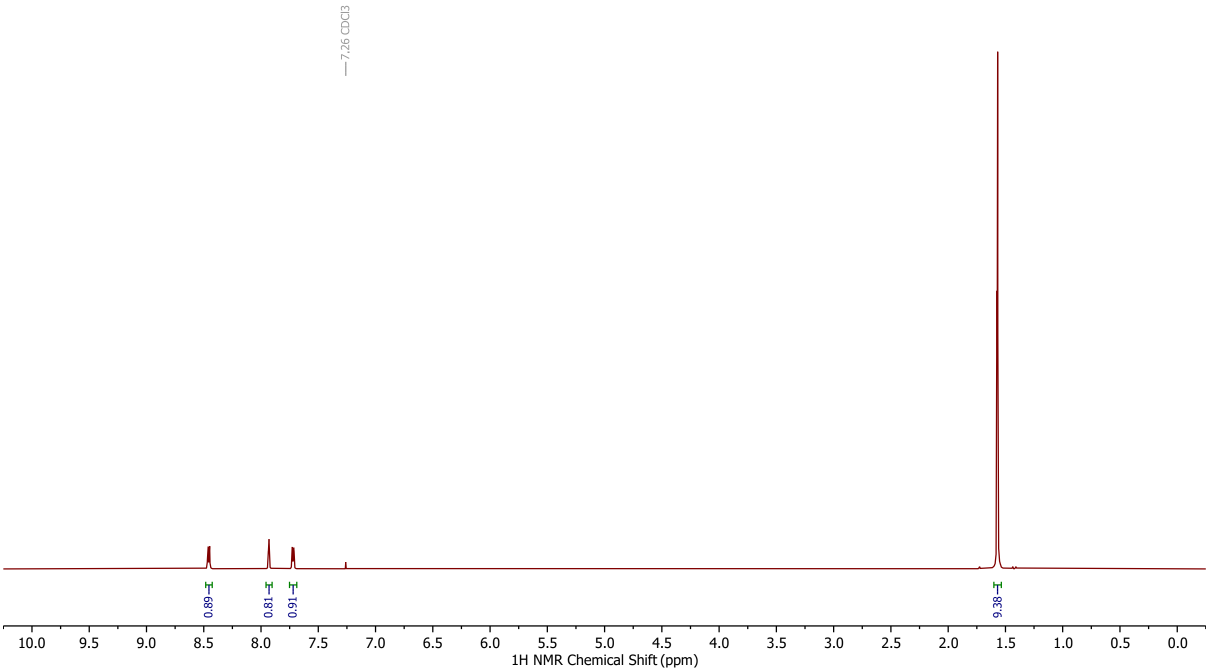

^{13}C NMR (126 MHz, 300 K, $CDCl_3$):

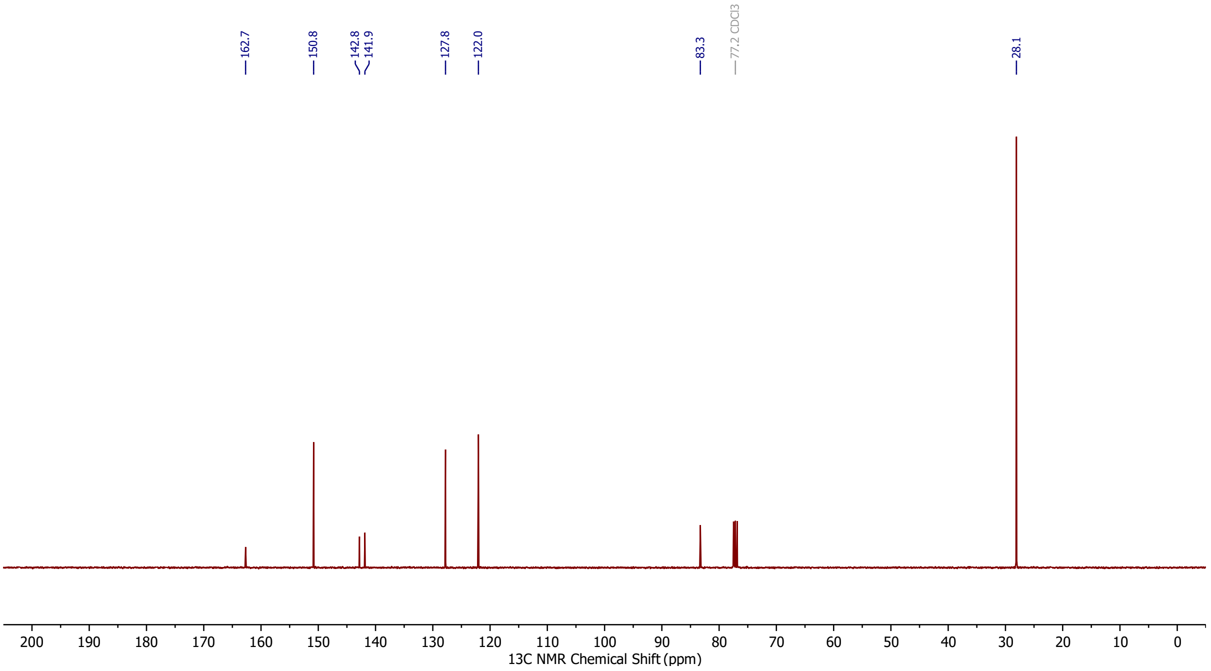

tert-Butyl 2-(1-methyl-1_H_-pyrazol-3-yl)isonicotinate (3)

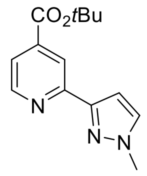

^{1}H NMR (600 MHz, 300 K, $CDCl_3$):

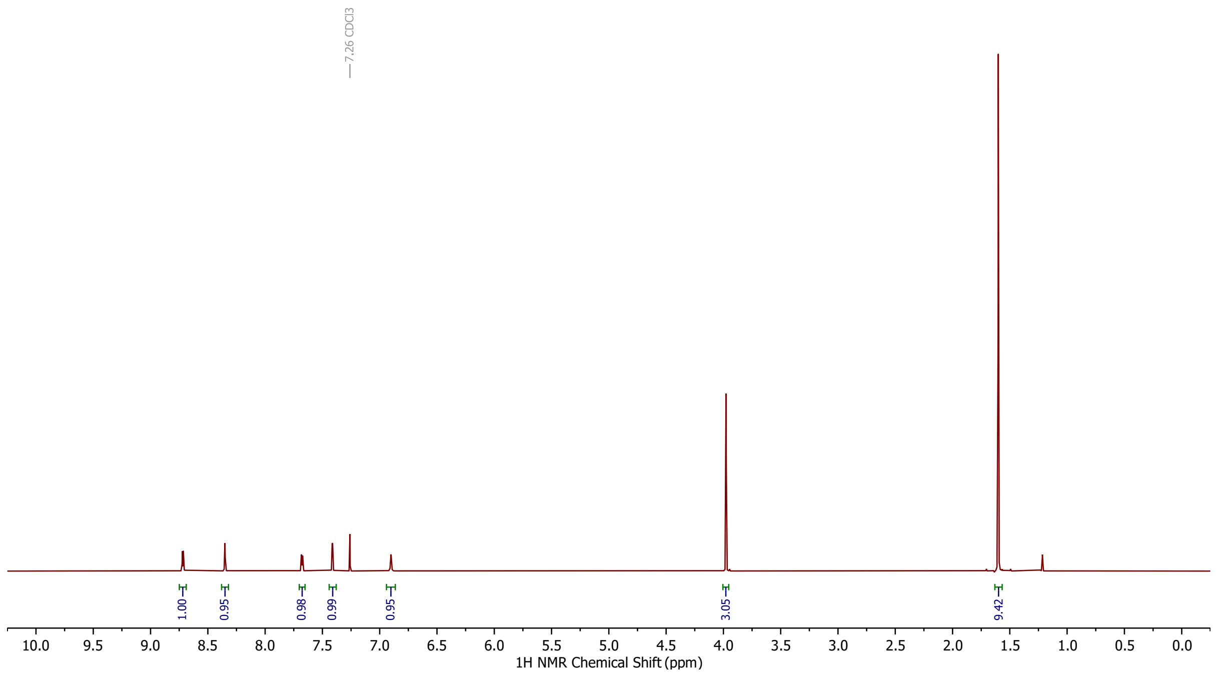

^{13}C NMR (151 MHz, 300 K, $CDCl_3$):

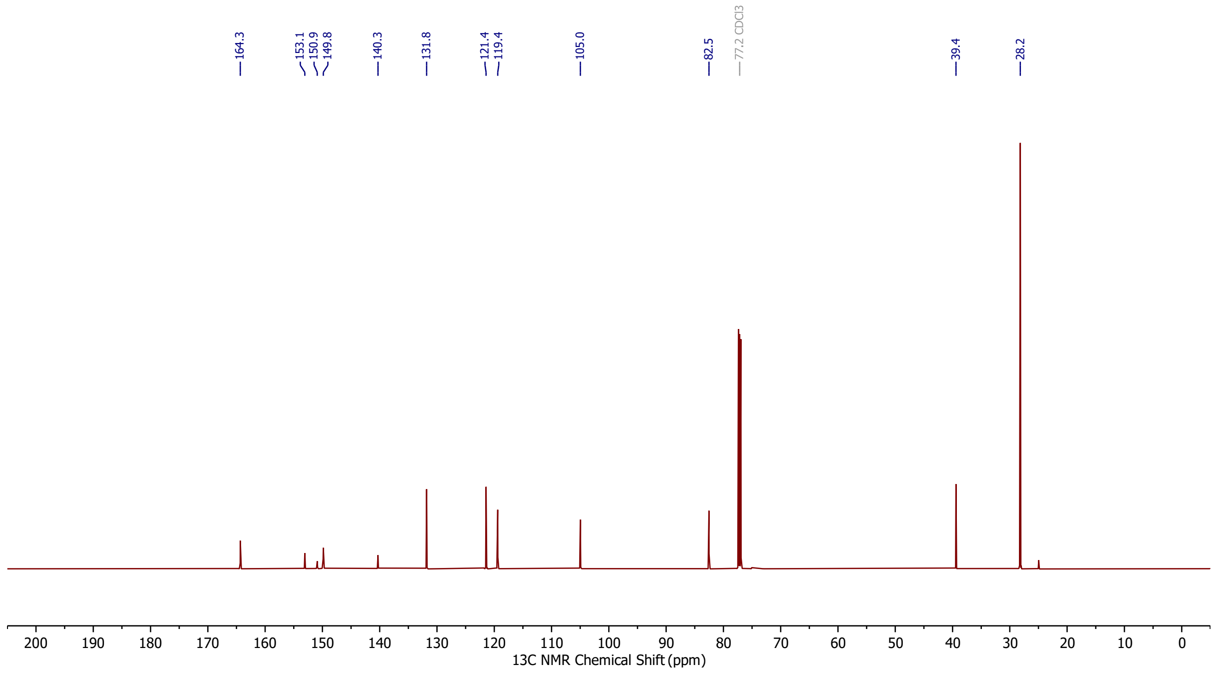

Compound 10b[7]

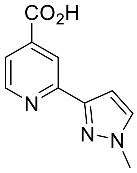

^{1}H NMR (600 MHz, 300 K, DMSO-d_6):

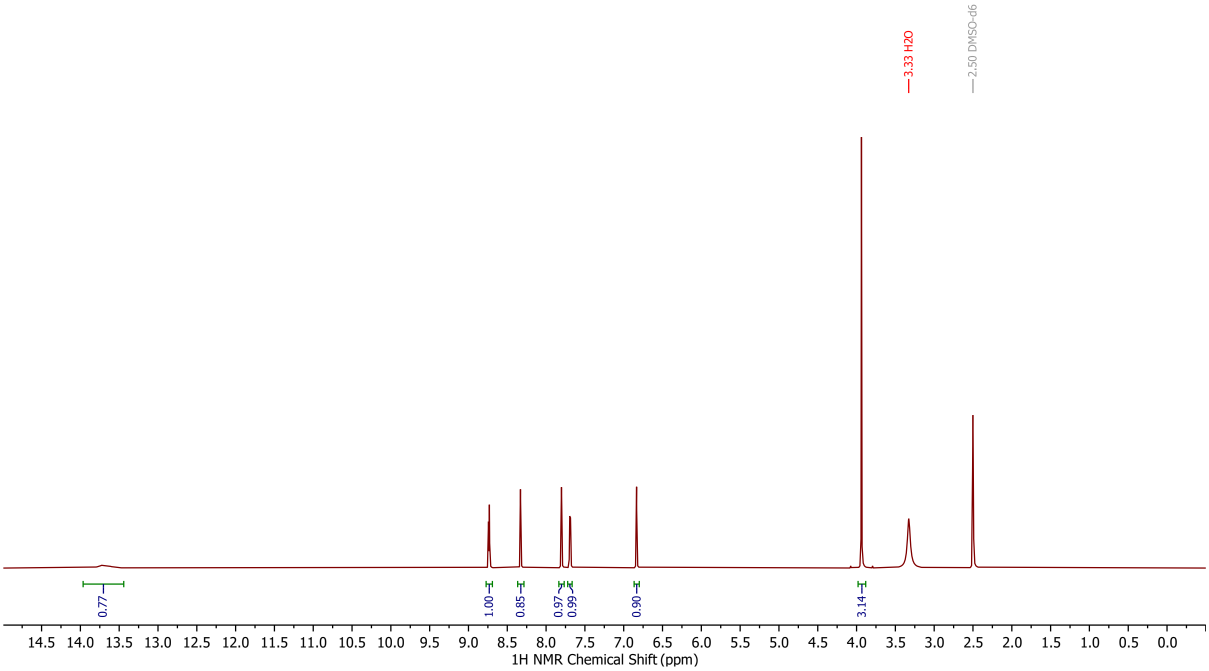

^{13}C NMR (151 MHz, 300 K, DMSO-d_6):

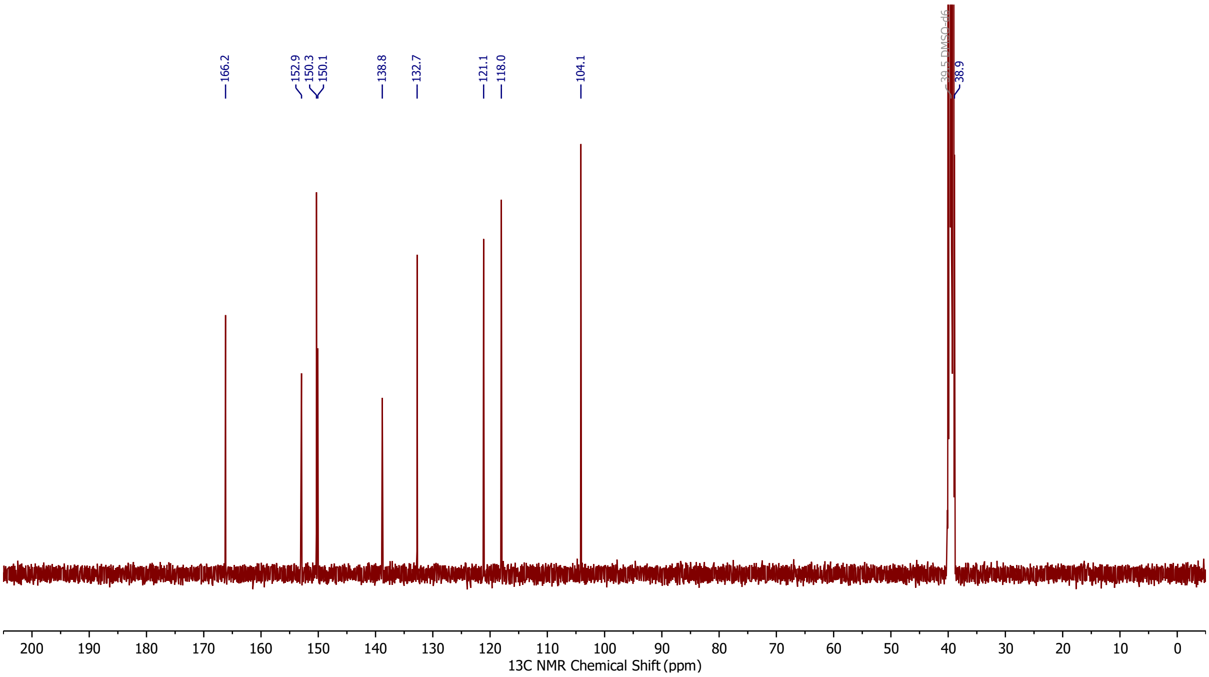

Compound 7p[7]

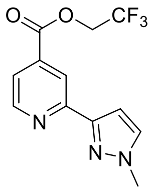

^1H NMR (600 MHz, 300 K, DMSO-d_6):

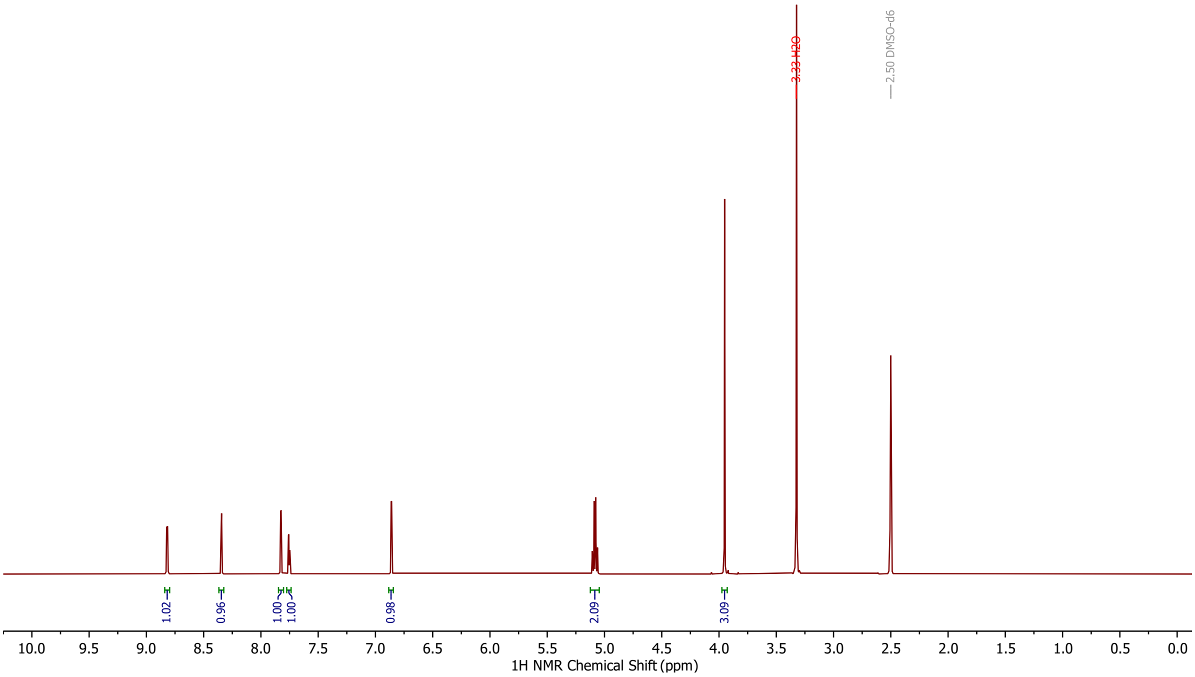

^{13}C NMR (151 MHz, 300 K, DMSO-d_6):

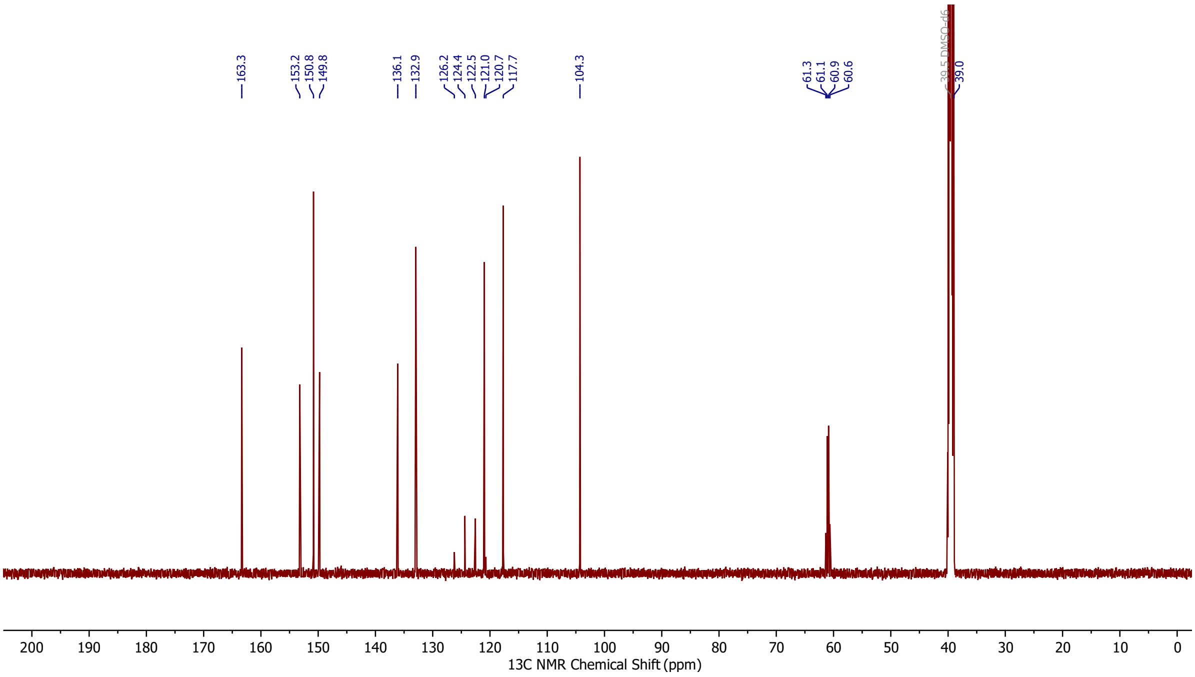

Methyl 3-((furan-2-ylmethyl)amino)isonicotinate (5)

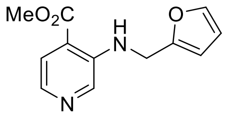

^{1}H NMR (600 MHz, 300 K, $CDCl_3$):

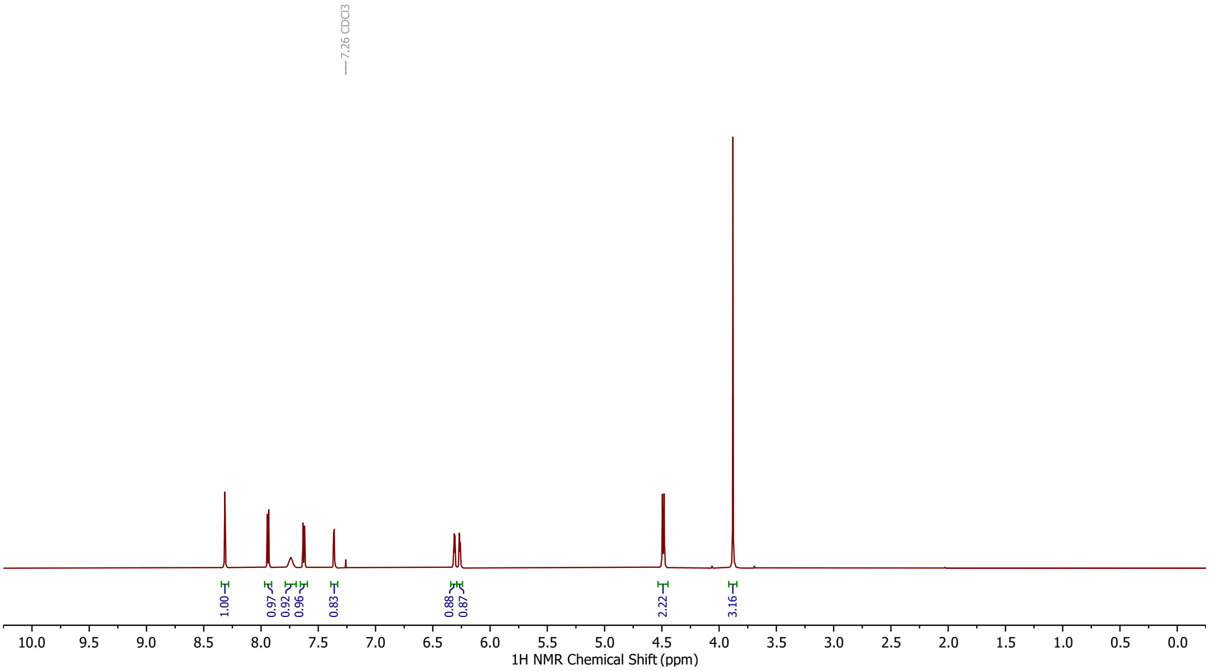

^{13}C NMR (151 MHz, 300 K, $CDCl_3$):

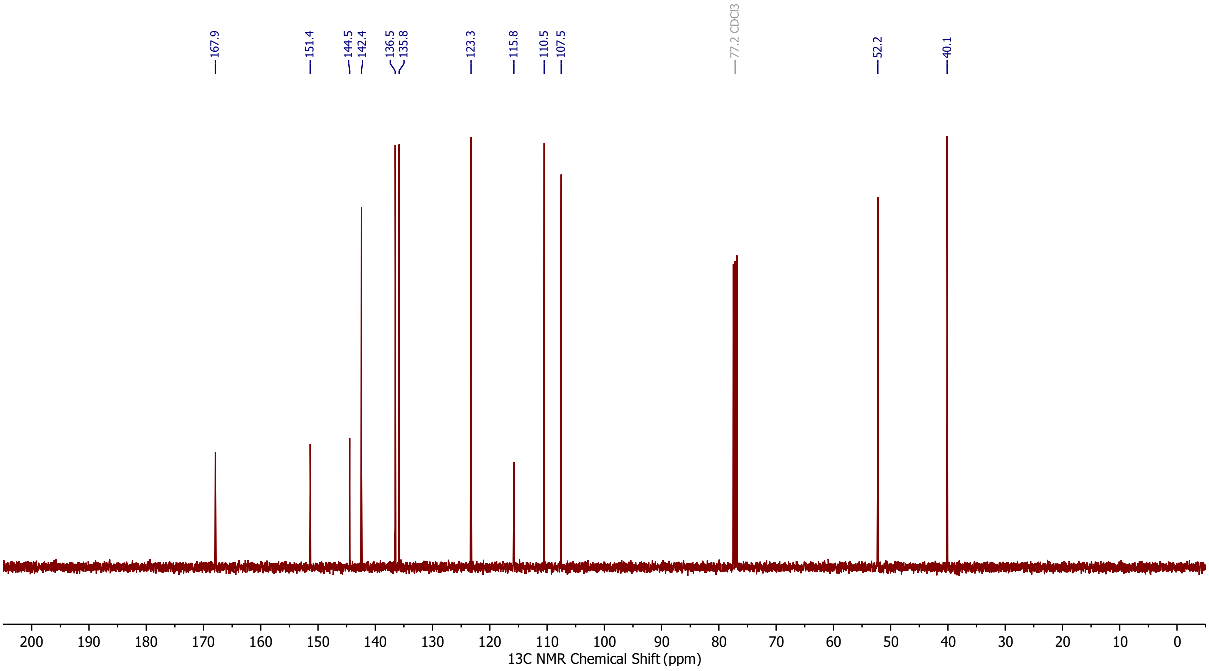

Compound 34[10]

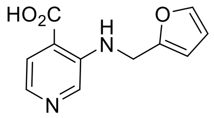

^{1}H NMR (600 MHz, 300 K, D_2O):

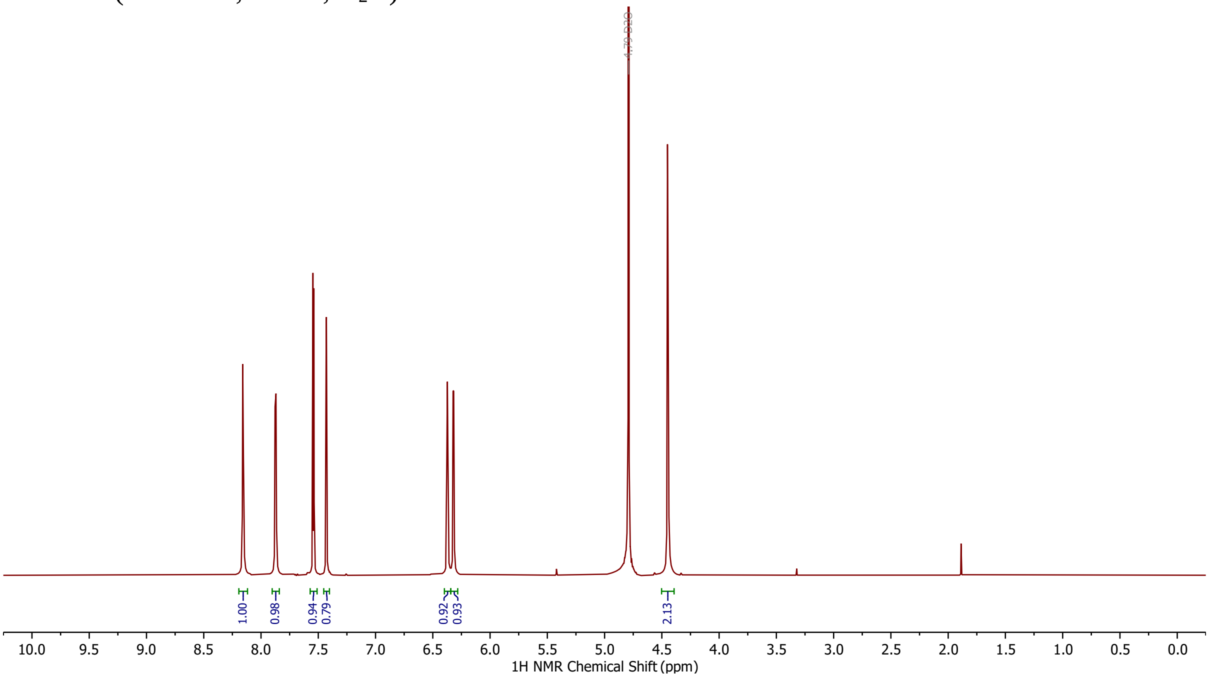

^{13}C NMR (151 MHz, 300 K, D_2O):

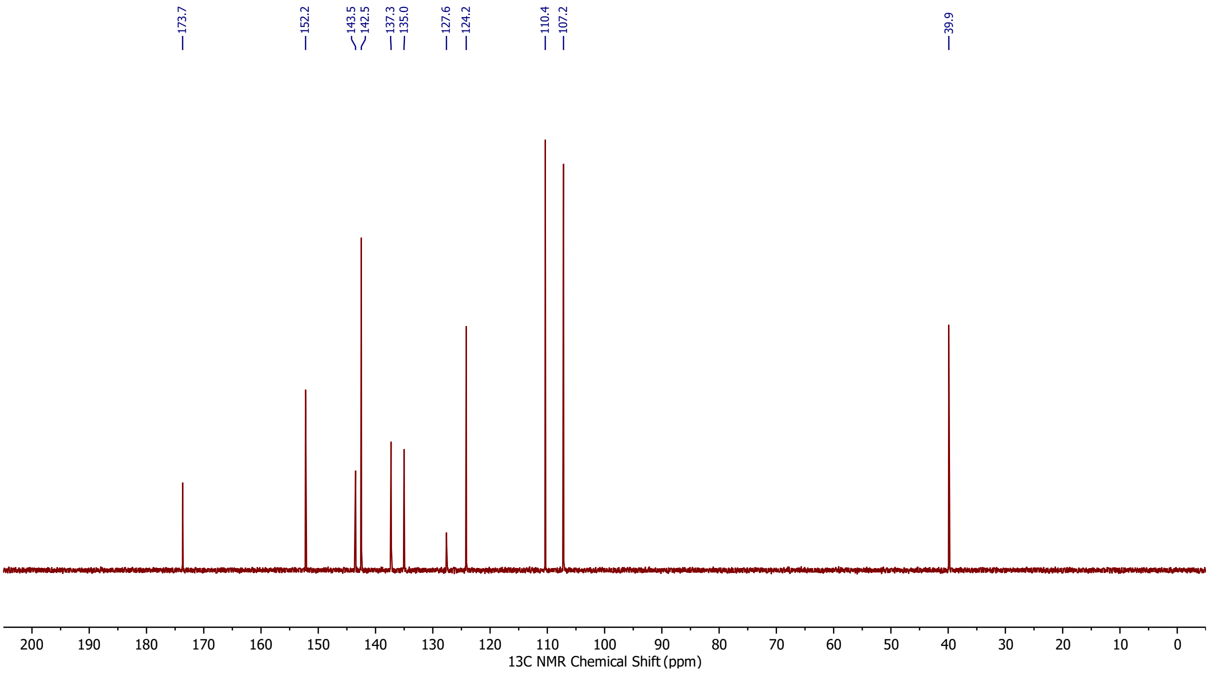

7. References

[1] J.-H. Zhang, T. D. Y. Chung, K. R. Oldenburg, *J. Biomol. Screen.* **1999**, *4*, 67–73.

[2] N. R. Rose, M. A. McDonough, O. N. F. King, A. Kawamura, C. J. Schofield, *Chem. Soc. Rev.* **2011**, *40*, 4364–4397.

[3] O. N. F. King, X. S. Li, M. Sakurai, A. Kawamura, N. R. Rose, S. S. Ng, A. M. Quinn, G. Rai, B. T. Mott, P. Beswick, R. J. Klose, U. Oppermann, A. Jadhav, T. D. Heightman, D. J. Maloney, C. J. Schofield, A. Simeonov, *PLoS One* **2010**, *5*, e15535.

[4] N. R. Rose, E. C. Woon, A. Tumber, L. J. Walport, R. Chowdhury, X. S. Li, O. N. King, C. Lejeune, S. S. Ng, T. Krojer, M. C. Chan, A. M. Rydzik, R. J. Hopkinson, K. H. Che, M. Daniel, C. Strain-Damerell, C. Gileadi, G. Kochan, I. K. H. Leung, J. Dunford, K. K. Yeoh, P. J. Ratcliffe, N. Burgess-Brown, F. von Delft, S. Muller, B. Marsden, P. E. Brennan, M. A. McDonough, U. Oppermann, R. J. Klose, C. J. Schofield, A. Kawamura, *J. Med. Chem.* **2012**, *55*, 6639–6643.

[5] T. Ran, R. Xiao, Q. Huang, H. Yuan, T. Lu, W. Liu, *ACS Med. Chem. Lett.* **2019**, *10*, 1609–1613.

[6] H. Zheng, Y. Tie, Z. Fang, X. Wu, T. Yi, S. Huang, X. Liang, Y. Qian, X. Wang, R. Pi, S. Chen, Y. Peng, S. Yang, X. Zhao, X. Wei, *Signal Transduct. Target Ther.* **2019**, *4*, 24.

[7] T. Wang, R. Zhang, Y. Liu, Z. Fang, H. Zhang, Y. Fan, S. Yang, R. Xiang, *Bioorg. Med. Chem. Lett.* **2021**, *44*, 128109.

[8] X. Luo, Y. Liu, S. Kubicek, J. Myllyharju, A. Tumber, S. Ng, K. H. Che, J. Podoll, T. D. Heightman, U. Oppermann, S. L. Schreiber, X. Wang, *J. Am. Chem. Soc.* **2011**, *133*, 9451–9456.

[9] G. Rai, A. Kawamura, A. Tumber, Y. Liang, J. L. Vogel, J. H. Arbuckle, N. R. Rose, T. S. Dexheimer, T. L. Foley, O. N. King, A. Quinn, B. T. Mott, C. J. Schofield, U. Oppermann, A. Jadhav, A. Simeonov, T. M. Kristie, D. J. Maloney, in *Probe Reports from the NIH Molecular Libraries Program*, National Center for Biotechnology Information (US), Bethesda (MD), **2010**.

[10] S. M. Westaway, A. G. S. Preston, M. D. Barker, F. Brown, J. A. Brown, M. Campbell, C.-w. Chung, H. Diallo, C. Douault, G. Drewes, R. Eagle, L. Gordon, C. Haslam, T. G. Hayhow, P. G. Humphreys, G. Joberty, R. Katso, L. Kruidenier, M. Leveridge, J. Liddle, J. Mosley, M. Muelbaier, R. Randle, I. Rioja, A. Rueger, G. A. Seal, R. J. Sheppard, O. Singh, J. Taylor, P. Thomas, D. Thomson, D. M. Wilson, K. Lee, R. K. Prinjha, *J. Med. Chem.* **2016**, *59*, 1357–1369.

[11] Y. K. Chen, T. Bonaldi, A. Cuomo, J. R. Del Rosario, D. J. Hosfield, T. Kanouni, S.-c. Kao, C. Lai, N. A. Lobo, J. Matuszkiewicz, A. McGeehan, S. M. O'Connell, L. Shi, J. A. Stafford, R. K. Stansfield, J. M. Veal, M. S. Weiss, N. Y. Yuen, M. B. Wallace, *ACS Med. Chem. Lett.* **2017**, *8*, 869–874.

[12] C. Chandhasin, V. Dang, F. Perabo, J. Del Rosario, Y. K. Chen, E. Filvaroff, J. A. Stafford, M. Clarke, *Anti-Cancer Drugs* **2023**, *34*, 1122–1131.

[13] N. Cao, Y. Huang, J. Zheng, C. I. Spencer, Y. Zhang, J.-D. Fu, B. Nie, M. Xie, M. Zhang, H. Wang, T. Ma, T. Xu, G. Shi, D. Srivastava, S. Ding, *Science* **2016**, *352*, 1216–1220.

[14] T. P. Corner, R. Z. R. Teo, Y. Wu, E. Salah, Y. Nakashima, G. Fiorini, A. Tumber, A. Brasnett, J. P. Holt-Martyn, W. D. Figg, X. Zhang, L. Brewitz, C. J. Schofield, *Chem. Sci.* **2023**, *14*, 12098–12120.

[15] a) C. M. Tegley, V. N. Viswanadhan, K. Biswas, M. J. Frohn, T. A. N. Peterkin, C. Chang, R. W. Bürli, J. H. Dao, H. Veith, N. Rogers, S. C. Yoder, G. Biddlecome, P. Tagari, J. R. Allen, R. W. Hungate, *Bioorg. Med. Chem. Lett.* **2008**, *18*, 3925–3928; b) M. C. Chan, N. E. Ilott, J. Schödel, D. Sims, A. Tumber, K. Lippl, D. R. Mole, C. W. Pugh, P. J. Ratcliffe, C. P. Ponting, C. J. Schofield, *J. Biol. Chem.* **2016**, *291*, 20661–20673.

[16] S. Kato, N. Ochiai, H. Takano, F. Io, N. Takayama, H. Koretsune, E.-i. Kunioka, S. Uchida, K. Yamamoto, *J. Pharmacol. Exp. Ther.* **2019**, *371*, 675–683.

[17] Y. Ogoshi, T. Matsui, I. Mitani, M. Yokota, M. Terashita, D. Motoda, K. Ueyama, T. Hotta, T. Ito, Y. Hase, K. Fukui, K. Deai, H. Yoshiuchi, S. Ito, H. Abe, *ACS Med. Chem. Lett.* **2017**, *8*, 1320–1325.

[18] D. V. Parmar, K. A. Kansagra, J. C. Patel, S. N. Joshi, N. S. Sharma, A. D. Shelat, N. B. Patel, V. B. Nakrani, F. A. Shaikh, H. V. Patel, ZYAN1 Trial Investigators, *Am. J. Nephrol.* **2019**, *49*, 470–478.

[19] T. P. Corner, A. Tumber, E. Salah, M. Jabbary, Y. Nakashima, L. I. Schnaubelt, S. Basak, F. M. Alshref, L. Brewitz, C. J. Schofield, *J. Med. Chem.* **2025**, *68*, 9777–9798.

[20] R. Chowdhury, J. I. Candela-Lena, M. C. Chan, D. J. Greenald, K. K. Yeoh, Y.-M. Tian, M. A. McDonough, A. Tumber, N. R. Rose, A. Conejo-Garcia, M. Demetriades, S. Mathavan, A. Kawamura, M. K. Lee, F. van Eeden, C. W. Pugh, P. J. Ratcliffe, C. J. Schofield, *ACS Chem. Biol.* **2013**, *8*, 1488–1496.

[21] J. L. Ariazi, K. J. Duffy, D. F. Adams, D. M. Fitch, L. Luo, M. Pappalardi, M. Biju, E. H. DiFilippo, T. Shaw, K. Wiggall, C. Erickson-Miller, *J. Pharmacol. Exp. Ther.* **2017**, *363*, 336–347.

[22] P. E. Pergola, B. S. Spinowitz, C. S. Hartman, B. J. Maroni, V. H. Haase, *Kidney Int.* **2016**, *90*, 1115–1122.

[23] H. Beck, M. Jeske, K. Thede, F. Stoll, I. Flamme, M. Akbaba, J.-K. Ergüden, G. Karig, J. Keldenich, F. Oehme, H.-C. Militzer, I. V. Hartung, U. Thuss, *ChemMedChem* **2018**, *13*, 988–1003.

[24] J. S. Debenham, C. Madsen-Duggan, M. J. Clements, T. F. Walsh, J. T. Kuethe, M. Reibarkh, S. P. Salowe, L. M. Sonatore, R. Hajdu, J. A. Milligan, D. M. Visco, D. Zhou, R. B. Lingham, D. Stickens, J. A. DeMartino, X. Tong, M. Wolff, J. Pang, R. R. Miller, E. C. Sherer, J. J. Hale, *J. Med. Chem.* **2016**, *59*, 11039–11049.

[25] R. Martin, S. L. Buchwald, *Acc. Chem. Res.* **2008**, *41*, 1461–1473.

[26] a) J. Louie, J. F. Hartwig, *Tetrahedron Lett.* **1995**, *36*, 3609–3612; b) A. S. Guram, R. A. Rennels, S. L. Buchwald, *Angew. Chem., Int. Ed.* **1995**, *34*, 1348–1350.

[27] P. C. Kamer, P. W. van Leeuwen, J. N. Reek, *Acc. Chem. Res.* **2001**, *34*, 895–904.

[28] M. S. Islam, M. A. McDonough, R. Chowdhury, J. Gault, A. Khan, E. Pires, C. J. Schofield, *J. Biol. Chem.* **2019**, *294*, 11637–11652.

[29] H. M. Berman, J. Westbrook, Z. Feng, G. Gilliland, T. N. Bhat, H. Weissig, I. N. Shindyalov, P. E. Bourne, *Nucleic Acids Res.* **2000**, *28*, 235–242.

[30] J. M. Word, S. C. Lovell, J. S. Richardson, D. C. Richardson, *J. Mol. Biol.* **1999**, *285*, 1735–1747.

[31] V. B. Chen, W. B. Arendall III, J. J. Headd, D. A. Keedy, R. M. Immormino, G. J. Kapral, L. W. Murray, J. S. Richardson, D. C. Richardson, *Acta Cryst.* **2010**, *D66*, 12–21.

[32] C. R. Søndergaard, M. H. Olsson, M. Rostkowski, J. H. Jensen, *J. Chem. Theory Comput.* **2011**, *7*, 2284–2295.

[33] W. L. DeLano, **2002**, De Lano Scientific, San Carlos.

[34] G. Jones, P. Willett, R. C. Glen, A. R. Leach, R. Taylor, *J. Mol. Biol.* **1997**, *267*, 727–748.

[35] M. L. Verdonk, J. C. Cole, M. J. Hartshorn, C. W. Murray, R. D. Taylor, *Proteins* **2003**, *52*, 609–623.

[36] H. Lawson, J. P. Holt-Martyn, V. Dembitz, Y. Kabayama, L. M. Wang, A. Bellani, S. Atwal, N. Saffoon, J. Durko, L. N. van de Lagemaat, A. L. De Pace, A. Tumber, T. Corner, E. Salah, C. Arndt, L. Brewitz, M. Bowen, L. Dubusse, D. George, L. Allen, A. V. Guitart, T. K. Fung, C. W. E. So, J. Schwaller, P. Gallipoli, D. O'Carroll, C. J. Schofield, K. R. Kranc, *Nat. Cancer* **2024**, *5*, 916–937.
